# Mechanistic insight into the assembly of the HerA–NurA helicase–nuclease DNA end resection complex

**DOI:** 10.1093/nar/gkx890

**Published:** 2017-10-09

**Authors:** Zainab Ahdash, Andy M. Lau, Robert Thomas Byrne, Katja Lammens, Alexandra Stüetzer, Henning Urlaub, Paula J. Booth, Eamonn Reading, Karl-Peter Hopfner, Argyris Politis

**Affiliations:** 1 Department of Chemistry, King's College London, 7 Trinity Street, London SE1 1DB, UK; 2 Gene Center and Department of Biochemistry, Ludwig-Maximilians-Universität München, Feodor-Lynen-Strasse 25, 81377 München, Germany; 3 Bioanalytical Mass Spectrometry Group, MPI for Biophysical Chemistry, D-37077 Göttingen, Germany; 4 Bioanalytics Group, Institute for Clinical Chemistry, University Medical Center Göttingen, D-37075 Göttingen, Germany

## Abstract

The HerA–NurA helicase–nuclease complex cooperates with Mre11 and Rad50 to coordinate the repair of double-stranded DNA breaks. Little is known, however, about the assembly mechanism and activation of the HerA–NurA. By combining hybrid mass spectrometry with cryo-EM, computational and biochemical data, we investigate the oligomeric formation of HerA and detail the mechanism of nucleotide binding to the HerA–NurA complex from thermophilic archaea. We reveal that ATP-free HerA and HerA-DNA complexes predominantly exist in solution as a heptamer and act as a DNA loading intermediate. The binding of either NurA or ATP stabilizes the hexameric HerA, indicating that HerA–NurA is activated by substrates and complex assembly. To examine the role of ATP in DNA translocation and processing, we investigated how nucleotides interact with the HerA–NurA. We show that while the hexameric HerA binds six nucleotides in an ‘all-or-none’ fashion, HerA–NurA harbors a highly coordinated pairwise binding mechanism and enables the translocation and processing of double-stranded DNA. Using molecular dynamics simulations, we reveal novel inter-residue interactions between the external ATP and the internal DNA binding sites. Overall, here we propose a stepwise assembly mechanism detailing the synergistic activation of HerA–NurA by ATP, which allows efficient processing of double-stranded DNA.

## INTRODUCTION

DNA double-stranded breaks (DSBs) are one of the most cytotoxic and deleterious forms of DNA damage which can result in genetic instability and can lead to the development of cancer in humans (1). DSBs can be repaired through either homologous recombination (HR) (2,3) or several end joining pathways (3,4).

In archaeal organisms, DNA resection requires the cooperation between the ATP-dependent Mre11–Rad50 and HerA–NurA complexes, which are encoded in the same operon (5–7). Several studies have demonstrated that these proteins are capable of working in concert *in vitro* to process dsDNA (8–10). HerA and NurA are considered as functional homologs of the eukaryotic Dna2/DNA2, Exo1/EXO1 and Sgs1/BLM proteins and therefore the assembled HerA–NurA system serves as a suitable model system for studying HR (11–15). During HR, DNA ends are resected through coordinated action of helicases and nucleases which unwind the DNA duplex and generate 3′ overhangs that are required for the subsequent repair steps (3). Several biochemical characterizations revealed that HerA is a helicase which exhibits ATPase activity while NurA is a 5′-3′ ssDNA/dsDNA exonuclease and ssDNA endonuclease (5,6,16,17). The helicase activity of HerA and nuclease activity of NurA are inter-dependent (7).

A recent crystallographic study revealed the high-resolution structure of HerA from thermophilic archaeon *Sulfolobus solfataricus*, co-crystalized with a non-hydrolysable ATP analog (AMP-PNP) (15). Within the crystallographic unit cell, six HerA monomers are assembled as a hexameric ring, which is stabilized through interaction of a conserved C-terminal brace between adjacent HerA protomers (15). The crystal structure of NurA shows the nuclease homodimer arranged in a toroidal configuration of RNaseH-Like domains by intertwining the helical N- and C-terminal extensions (7,10). NurA dimer has a central channel lined with opposed active sites. The inner surface of mainly positively-charged residues facilitates interactions with the phosphodiester chains of DNA (7). The central channel of NurA is capable of accommodating up to two strands of unwound DNA (7).

The HerA–NurA complex is composed of a homohexameric HerA helicase and a homodimeric NurA nuclease. The topology of the assembled HerA–NurA is further detailed by a recent cryo-electron microscopy (cryo-EM) density map, which shows the interaction of HerA with NurA to form a continuous channel through both HerA and NurA (18). HerA–NurA couples chemical energy from the hydrolysis of nucleotide triphosphates (NTP) such as ATP, to mechanical translocation along the DNA substrates (15,19,20). Despite the availability of structural information, a detailed mechanistic understanding of how complex assembly and nucleotide binding can coordinate the conformational changes that lead to a functional machine are currently limited. To address these issues, we have carried out a detailed biochemical and biophysical characterization of the HerA–NurA complex, using native mass spectrometry (MS).

Native MS has emerged as a key tool that can interrogate the structure and dynamics of large and heterogeneous protein assemblies (21–25). Mass spectrometric analysis of biomolecules can be coupled with nano electrospray ionisation (nESI) and can preserve the structure and non-covalent interactions of proteins in near-native solution state configurations (21,26). The introduction of protein assemblies into the gas phase and its coupling with ion mobility mass spectrometry (IM-MS) has enabled characterization of the overall topology, conformational state and dynamics of proteins and their complexes. Impressive applications of native MS and IM-MS include the generation of protein-protein connectivity networks in macromolecular complexes (27–32), the monitoring of their assembly pathways (33–37) and the interactions between proteins and nucleic acids (38–44).

In the present study, we dissect the mechanism of action of the HerA–NurA complex. We show that the nucleotide-free HerA in solution is in equilibrium between hexameric and heptameric oligoforms, the latter being largely dominant. We show that while hexameric HerA can bind dsDNA regardless of nucleotide binding, the heptameric form can only bind dsDNA in the absence of nucleotides. We highlight a major role of NurA as the assembly organizer, which selects hexameric HerA to form the HerA–NurA resection complex. In addition, hybrid MS in combination with molecular dynamics (MD) simulations, cryo-EM and biochemical assays allow deconvolution of the nucleotide binding modality of HerA–NurA, and reveal a non-concerted mechanism of nucleotide binding. We show that the HerA helicase of HerA–NurA utilises ATP hydrolysis in coordinated pairs, driving dsDNA translocation and unwinding. Combining structural and functional data, we provide new molecular insights and propose a model of the assembly mechanism of the HerA–NurA DNA end-resection complex, leading to DNA translocation and processing.

## MATERIALS AND METHODS

### Protein purification

HerA and NurA cells were re-suspended in lysis buffer (100 mM NaCl, 50 mM Hepes–NaOH pH 8.0, 5 mM DTT and 5% v/v glycerol) supplemented with an EDTA-Free SigmaFast Protease Inhibitor Cocktail Tablet (Sigma-Aldrich). The cells were lysed by sonication and the lysate was clarified by centrifugation. The supernatant was heated in a 70°C water bath for 20 min and subsequently clarified by centrifugation to remove precipitated protein. The supernatant was then loaded onto a 5 ml Heparin HP column (GE Healthcare) equilibrated with lysis buffer. Bound protein was eluted with a linear gradient from 100 mM to 1 M NaCl. Fractions containing the protein of interest were pooled and dialysed against 100 mM NaCl, 50 mM Hepes–NaOH pH 8.0, 5 mM DTT and 5% (v/v) glycerol for 18 h. The dialyzed protein was loaded onto a 5 mL HiTrap Q HP column (GE Healthcare) equilibrated with lysis buffer. Bound protein was eluted with a linear gradient from 100 mM to 1 M NaCl. Fractions containing the protein of interest were pooled and dialysed against 300 mM NaCl, 20 mM Hepes–NaOH pH 8.0, 5 mM DTT and 5% (v/v) glycerol for 18 h. The protein was concentrated with Amicon Ultra-15 centrifugal filter units (EMD Millipore) and aliquots were flash-frozen in liquid nitrogen and stored at –80°C. Protein concentrations were estimated from absorbance at 280 nm using extinction coefficients.

### Mass spectrometry

Twenty microliter of purified protein was buffer exchanged into MS buffer (200 mM ammonium acetate, at pH 7.0–7.5) using Vivaspin^®^500 centrifugal concentrator (GE Healthcare). A total of 6–8 washes were performed. Protein concentrations used are reported in each relevant section. For DNA bound complexes, protein and a 25 bp dsDNA (5′-GTAGTCCGGACGACAAACGCCGACT-3′) were mixed at a 1:1 molar ratio and incubated for 30 min at room temperature. For MS analysis, 2–3 μl aliquots of the sample was directly infused via nano electrospray using gold-coated borosilicate capillaries. All spectra were recorded on a Synapt G2Si High Definition MS system (Waters). Instrument settings were 1.4–1.7 kV capillary voltage, 20–60 V for cone voltage, 30–50 V for collision voltage, extraction voltage of 8 V and a transfer voltage of 2 V. The source pressure was 5–7 mbar. The bias voltage was 30–35 V. Drift cell gas was N2 (pressure of 1.6 Torr) and the collision gas was argon with flow rate set to 5–8 ml min^–1^. Helium was used as the buffer gas at 2.1 Torr. Source temperature was 20–25°C. The relative intensities of species were calculated using the UniDec deconvolution software (45), which takes into account the detector efficiency (46). IM-MS experiments were performed at a capillary voltage of 1.4–1.7 kV, capillary nanoflow of 0.01–0.05 mBar, collision voltage 30 V and a cone voltage of 40 V. Triplicate measurements of mobility separation were performed by keeping a constant wave height (40 V) and varying the wave velocity (540–640 ms^−1^). Replicate measurements were performed under identical instrumental conditions. IM arrival time distributions (ATDs) were extracted and converted into ^TW^CCS_He_ distributions using a calibration of protein standards, avidin (64 kDa), concanavalin A (103 kDa), alcohol dehydrogenase (143 kDa), glutamate dehydrogenase (GDH, 336 kDa) and beta-galactosidase (464 kDa). The model CCS was calculated from respective crystal structure coordinates using the projection approximation (PA) method calculated using IMPACT software (http://impact.chem.ox.ac.uk/). The PA was then scaled for discrepancies between expected mass (M_exp_; from UniProt sequence), and mass of pdb model (M_pdb_) equation 1 (23,47).

(1) \begin{equation*}{\Omega _{{\rm{model}}}} = 1.14\Omega {({M_{{\rm exp}}}/{M_{{\rm pdb}}})^{2/3}}\end{equation*}

### Electron microscopy

Wild type HerA was diluted to a concentration of 40 μM in 300 mM NaCl, 20 mM Hepes–NaOH pH 8.0, 5 mM DTT and 5% (v/v) glycerol in a total volume of 150 μl. The sample was heated to 60°C for 20 min and centrifuged at 21 000 × g for 15 min. The supernatant was then loaded onto a Superdex 200 5/150 gel filtration column equilibrated with 100 mM NaCl and 20 mM Hepes–NaOH pH 8.0. The peak fraction was diluted to a concentration of 0.25 mg/ml in 100 mM NaCl, 20 mM Hepes–NaOH and 0.001% (v/v) Nonident P40 (Sigma-Aldrich). 4.5 μl of the diluted protein was pipetted onto a glow-discharged R 2/1 grid (Quantifoil). The sample was then vitrified in liquid ethane using an EM GP Automatic Plunge Freezer (Leica Microsystems) using a temperature of 15°C, relative humidity of 95%, no pre-blot incubation and a blotting time of 2 s (supplementary materials and methods).

### ATP and ADP titration using MS

HerA, HerA–NurA or HerA–NurA–dsDNA were mixed with increasing concentrations of the non-hydrolysable ATP analog adenosine 5′-*O*-(3′thiotriphosphate), tetralithium salt (ATP-γ-S) (Merck Millipore) and adenosine 5′-diphosphate (ADP) (Sigma-Aldrich) and 2 mM MgCl_2_ (Supplementary Table S3). Protein concentrations used are reported for each relevant experiment. Samples were incubated at room temperature for 1 h and then buffer-exchanged 6–8 times into 200 mM AA (pH 7–8) using a Vivaspin concentrator (Sartorius™) prior to analysis by MS. Three independent measurements were performed.

### ATP Hydrolysis Assay

The rates of ATP hydrolysis by HerA in the presence or absence of either NurA or dsDNA were measured using EnzChek^®^ Phosphate Assay Kit (Thermo Fisher Scientific). To reduce the effect of inorganic phosphate contaminations, we performed a ‘Pi mop’ as described in the manufacturer protocol. The protein was pre-incubated for an hour at room temperature in the assay reaction mixture before adding adenosine 5′-triphosphate magnesium salt (Sigma-Aldrich) and recording measurements at 360 nm for 7–8 min. The protein concentration was 100 nM and the ATP concentrations were ranging from 2 to 100 μM.

### Fluorescence anisotropy

HerA–NurA^H306A^ complexes were further purified as described above (protein purification) except that the Superdex 200 5/150 gel filtration column was equilibrated with 100 mM NaCl and 20 mM Hepes–NaOH pH 8.0. HerA was used without additional purification. For each titration 100 μl samples were prepared containing between 0–200 nM (HerA–NurA^H306A^) or 0–5000 nM (HerA) and 5 nM dsDNA with a 5′-fluorescein label in 100 mM NaCl and 20 mM Hepes–NaOH pH 8.0. Samples containing nucleotides additionally contained 2 mM of the nucleotide (either ADP or the non-hydrolysable analog ATP-y-S) and 5 mM MgCl_2_. All samples were prepared in 96 well chimney black microplates (Greiner) and incubated for 30 min at room temperature. Anisotropy values were measured with a M1000 Infinite plate reader (Tecan) using an excitation wavelength of 470 nm and an emission wavelength of 520 nm. Titrations were performed in triplicate. Data for HerA were fit in *Prism 5* (GraphPad Software) using the following single-site binding model:

\begin{equation*}{{y}}\ = {{{A}}_{{f}}}\ - \left( {{{{A}}_{{f}}} - {{{A}}_{{b}}}} \right) \cdot \frac{{{x}}}{{{{x}} + {{{K}}_{{D}}}}}\end{equation*}

where *y* is the observed anisotropy, *A*_*f*_ and *A*_*b*_ are the anisotropy values of the free and bound dsDNA (respectively), *x* is the protein concentration and *K_D_* is the dissociation constant. Data for HerA–NurA^H306A^ were fit similarly using the Hill equation:

\begin{equation*}{{y}}\ = {{{A}}_{{f}}}\ - \left( {{{{A}}_{{f}}} - {{{A}}_{{b}}}} \right) \cdot \frac{{{{{x}}^{{h}}}}}{{{{{x}}^{{h}}} + {{{K}}_{{D}}}}}\end{equation*}

where *h* is the Hill coefficient. The error bars shown in the figures are the standard error of the mean.

### Molecular modeling of the HerA–NurA

The DNA-bound variants of HerA–NurA, hexameric HerA and heptameric HerA were generated using PyMOL (www.pymol.org). A 25 bp DNA molecule of sequence 5′-d(GCTCCGATATTACAGTTGTAATTTT)-3′ was extracted from a MecI–DNA transcription repressor complex from *Staphylococcus aureus* (PDB ID: 1SAX). Overhanging 5′ bases were removed in order to generate a blunt double-stranded DNA molecule of 24 bp. The 24 bp DNA molecule was added to the cavity of HerA–NurA to yield the HerA–NurA–DNA complex. Hexameric HerA–DNA was generated through removing NurA from the full assembly. Heptameric HerA–DNA was generated through superimposing the heptameric HerA onto the hexameric HerA–DNA structure, and removing the hexameric helicase while preserving the position of DNA. ATP-bound HerA–NurA-DNA was assembled using the crystallographically available dimeric HerA-ANP asymmetric unit (PDB ID 4D2I). The hexameric ANP-bound barrel was formed via the aforementioned method. ANP ligands were converted to ATP through exchanging the γ-nitrogen atom for oxygen. The positions of all magnesium ions were preserved. The HerA–NurA-DNA-ATP assembly was formed exchanging the HerA-DNA complex for a HerA-DNA-ATP hexamer. The structure of HerA–NurA was assembled through rigid-body docking of the HerA (PDB 4D2I) and NurA (PDB 2YGK) crystal structures into a 7.35 Å cryo-EM density map of HerA–NurA (EMD-2808). The heptameric model of HerA was built using the existing hexameric HerA (PDB ID: 4D10). The six chains of the HerA hexamer were separated and each protomer was translated and rotated in order to build a heptameric ring with six subunits. The remaining seventh subunit was added through duplicating one HerA protomer.

### MD simulations in explicit solvent

A total of seven equilibrium simulations were ran for the HerA ± DNA (hexameric and heptameric), HerA–NurA ± DNA and HerA–NurA–DNA–6ATP–Mg structures. For all simulations, atomistic protein structures were added to a triclinic simulation box with a minimum distance of 1.0 nm between the edge of the periodic box and any protein atom. The CHARMM36 forcefield was used and TIP3P water molecules were added along with sodium ions in order to neutralize the system charge. Energy minimization utilized the steepest-descent algorithm for 50 000 iterations. Equilibration in the isochoric-isothermal ensemble was performed for 1 ns at 300 K with temperature time coupling (τ) of 0.1 ps. System temperatures were regulated by employing a velocity-rescaled Berendsen thermostat. All bonds were constrained using the LINCS algorithm. Next, the system was equilibrated in the isobaric-isothermal ensemble for 1ns at constant temperature of 300 K and pressure of 1 bar, enforced using the Berendsen thermostat (τ = 0.1 ps) and Parrinello-Rahman barostat (τ = 2 ps). Production simulation of the system was run for 50 ns at constant temperature and pressure. Long-range electrostatics were modeled using particle mesh Ewald electrostatics (grid spacing of 0.16 nm) with cubic interpolation. Van der Waals interactions were calculated using a twin range cut-off scheme and Lennard–Jones potential cut-off of 1.4 nm. All simulations and post-production analysis was performed using the GROMACS (www.gromacs.org) analysis tools.

## RESULTS

### HerA predominantly exists as a heptamer

**Figure 1.**
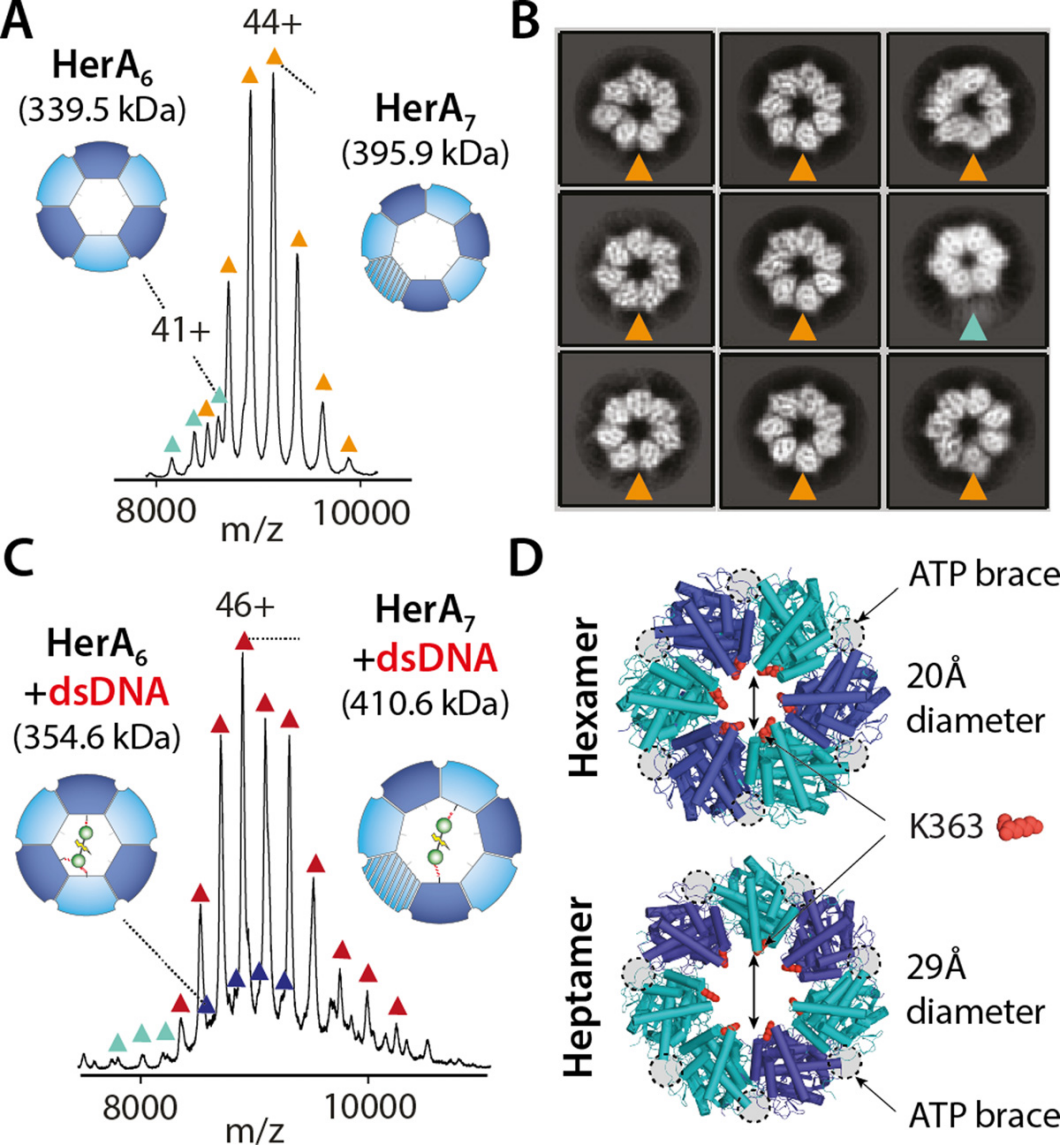
HerA co-exists as a hexamer and a heptamer. (**A**) Mass spectra shows higher abundance of the HerA heptamer. (**B**) 2D class averages from cryo-EM show that the HerA is primarily a heptamer. (**C**) Mass spectra of HerA–dsDNA. Both the hexamer and heptamer bind to dsDNA, where the heptamer is the main species. (**D**) Crystal structure of the HerA hexamer and model of the heptamer show the larger cavity of the heptamer. Grey circles highlight the nucleotide binding sites. The DNA-binding loop lysine K363 is shown in red (Supplementary Figure S1A).

To obtain information on the assembly pathway of the HerA–NurA, we subjected the wild-type apo-HerA complex to native MS. Well-resolved mass spectra revealed homo-hexameric and homo-heptameric oligoforms of HerA within solution (Figure 1A). To our surprise, our native MS analysis shows that heptameric HerA presides as the dominant oligoform and is approximately ten-fold more abundant than its hexameric counterpart. This is further corroborated by two-dimensional electron microscopy (EM) class averages, obtained from cryo-EM on the apo-HerA complex, that show an abundance of heptameric HerA (Figure 1B and Supplementary Figure S1A).

Next, to assess the ability of nucleotide-free HerA in binding dsDNA, we performed MS experiments on HerA following incubation with 25 base-pair (bp) blunt-ended dsDNA. While both hexameric and heptameric HerA can bind dsDNA (Figure 1C), doing so results in no change to the equilibrium of hexameric to heptameric HerA. Comparison of the crystallographic hexamer with our model of heptameric HerA revealed that the heptamer harbors a greater cavity diameter and thus provides a more accessible channel to dsDNA (Figure 1D). According to these observations, we therefore postulate that the heptameric HerA is topologically more efficient in binding to dsDNA substrates.

### Nucleotide binding converts heptameric to hexameric HerA

**Figure 2.**
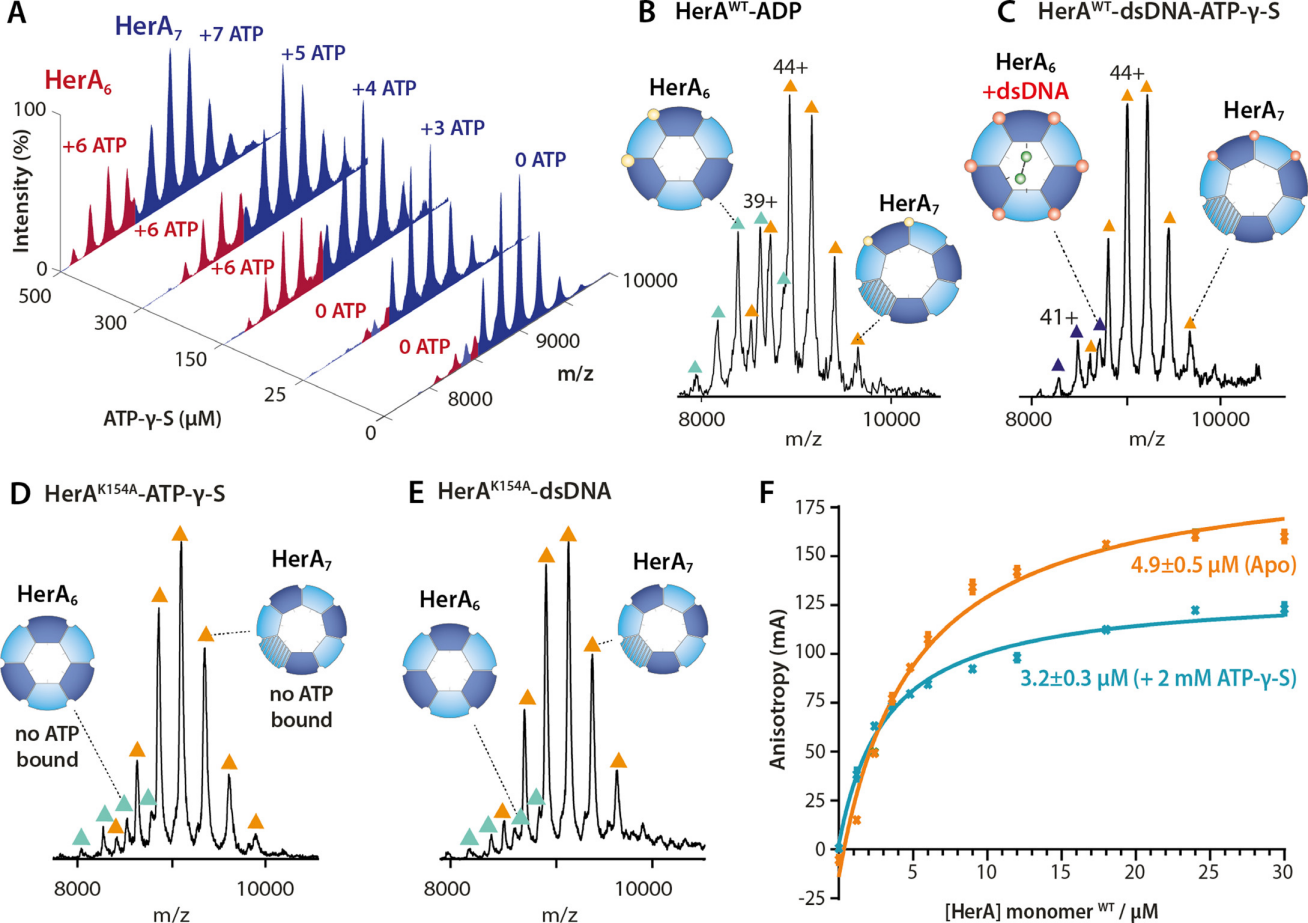
The effect of nucleotide binding on HerA oligomer formation and dsDNA binding. (**A**) Mass spectra of HerA at increasing concentrations of ATP-γ-S show increase of the hexameric HerA specie. ATP binds to HerA hexamers in an ‘all-or-none’ fashion; while it's binding to HerA heptamers follow a sequential manner. The titration was performed using 30μM of monomeric HerA. (**B**) Mass spectra of HerA obtained at a 1 mM ADP. (**C**) Mass spectra of dsDNA-bound HerA in the presence of 1mM ATP-γ-S. The catalytically inactive HerA^K154A^ does not bind (**D**) ATP-γ-S nor (**E**) dsDNA. (**F**) Fluorescence anisotropy assay measures the affinity of dsDNA (25 bp) to HerA in the presence and absence of a ATP-γ-S. Dissociation constants are shown (Supplementary Figure S1C-D and Supplementary Table S1).

We then examined the effect of nucleotide binding on the HerA oligoforms. Using a non-hydrolyzable ATP analog, ATP-γ-S, we were able to capture the ATP-bound HerA complexes. Increasing ATP-γ-S concentrations triggered substantial conversion of heptameric to hexameric HerA (Figure 2A). Overall, we measured a 4-fold increase in the intensity of hexameric HerA from ∼9% (no ATP-γ-S) to 37%, following saturation of all ATP binding sites (Figure 2A and Supplementary Figure 1B). A similar increase in hexameric HerA population was found when titrating HerA with increasing concentrations of ADP (Figure 2B). Furthermore, we observed that while the heptameric HerA binds ATP-γ-S in a sequential manner, the HerA hexamer binds all six ATP-γ-S simultaneously, in a with a ‘all-or-none’ mechanism of binding (Figure 2A). This difference in ATP-γ-S receptivity is likely to implicate local conformational changes and different mechanistic roles for ATP hydrolysis between the hexameric and heptameric forms.

In line with our previous experiments on nucleotide bound HerA, we then studied how dsDNA binds to HerA in the presence of nucleotides. In contrast to the nucleotide-free experiments, dsDNA could only bind to hexameric HerA (Figure 2C and Supplementary Figure S1C). As a control, we used the ATPase-inactive HerA K154A mutant (in the P-loop of the Walker A motif) that perturbs ATP binding (15). As expected, no nucleotide binding was observed with the K154A mutant HerA under identical experimental conditions (Figure 2D and Supplementary Figure S1D). Moreover, we discovered that the K154A mutation also perturbs dsDNA binding (Figure 2E), suggesting that functional ATP binding sites are required for dsDNA association regardless of whether or not they are occupied. We then measured the binding affinities of HerA for dsDNA in the presence and absence of ATP-γ-S using fluorescence anisotropy. The results showed that in the presence of ATP-γ-S, HerA binds to dsDNA with a minor increase in affinity (*K*_d_ = 3.2±0.3 μM) than the nucleotide-free HerA (*K*_d_ = 4.9 ± 0.5 μM) (Figure 2F and Supplementary Table S1). We attribute this difference to the ∼30% increase of the HerA hexamer populations in the presence of ATP-γ-S. The preferential binding of dsDNA to hexameric HerA in the presence of nucleotides, observed with native MS, suggests that the complex undergoes structural rearrangements upon nucleotide binding that favors the formation of the hexameric HerA–dsDNA complex. Together, these results suggest coordination between the ATP and dsDNA binding events in HerA.

### NurA selectively binds the HerA hexamer

**Figure 3.**
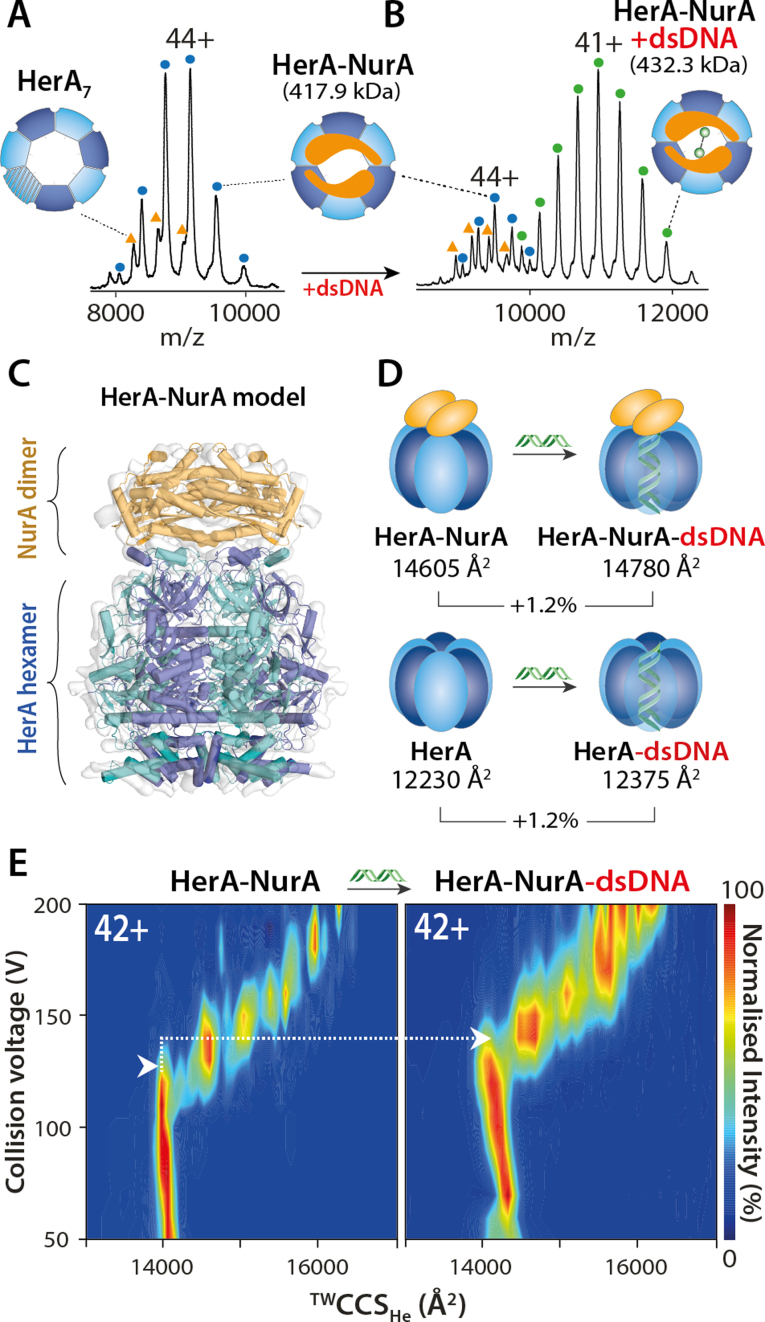
NurA selects the HerA hexamer to assemble the HerA–NurA complex. (**A**) NurA dimer binding to HerA causes oligomeric switch from heptameric to hexameric HerA. (**B**) dsDNA binds the hexameric HerA–NurA complex. (**C**) Building of the HerA–NurA assembly by fitting the crystal structures of the NurA dimer (PDB 2YGK) and HerA hexamer (PDB 4D2I) into the EM density map (EMD-2808) of the HerA–NurA assembly. (**D**) Average ^TW^CCS_He_ show that the binding of dsDNA to HerA hexamer or HerA–NurA increases by only 1.2% (average ^TW^CCS_He_ values shown) and supports dsDNA binding to the central HerA cavity. (**E**) Comparison of collision-induced unfolding (CIU) plots between HerA–NurA and HerA–NurA-dsDNA complexes. White arrows highlight the transition from the native-like to the first intermediate conformation. The HerA–NurA-dsDNA complex is more stable and requires more energy to unfold (Supplementary Figure S2 and Supplementary Table S2).

To determine the role of NurA in the assembled complex, we incubated HerA with NurA (2:3 molar ratio of monomers) in both the absence and presence of dsDNA. To access the HerA–NurA–dsDNA conformational states, we generated a nuclease-inactive NurA mutant H306A that prevents dsDNA degradation. In the absence of dsDNA, we observed that the NurA dimer imposes an oligomeric switch by selectively binding to the hexameric HerA and significantly depleting the heptameric population (Figure 3A and Supplementary Figure S2A). In the presence of dsDNA we found that NurA only binds to the hexameric HerA–dsDNA complex in line with the DNA-free measurements (Figure 3B). The oligomeric switch imposed by NurA on HerA in the presence of dsDNA is now accompanied by the complete disappearance of the heptameric HerA–dsDNA species (Figure 3B and Supplementary Figure S2A). Next, we determined the affinity of HerA–NurA^H306A^ for dsDNA. In comparison with HerA alone (Figure 2F), the affinity of nucleotide-free HerA–NurA^H306A^ for dsDNA was higher (K*_d_* = 510±30.4 nM) (Supplementary Figure S2B). Overall, the NurA dimer acts as an organizer of the complex that tightly binds to the hexameric HerA and enhances the affinity of the HerA–NurA for dsDNA.

### IM-MS reveals accommodation of dsDNA into the HerA–NurA cavity

Having established the assembly of HerA–NurA, we used ion mobility mass spectrometry (IM-MS) to detect potential conformational changes upon dsDNA binding. From IM-MS, the experimental CCS (^TW^CCS_He_), describing the rotationally-averaged cross-section of a protein can be calculated and compared with theoretical models (CCS_model_). To compliment IM-MS data, a model of the HerA–NurA assembly was built through rigid body docking of the crystallographic HerA and NurA into a cryo-EM density map (Figure 3C) (Methods). We further tested if our model accurately represents the solution-state structure of HerA–NurA by performing chemical crosslinking (XL) followed by MS (XL-MS) experiments (48) (Supplementary Figure S2C and D and Supplementary Table S2). For the apo-HerA–NurA complex, we observed a good agreement between the ^TW^CCS_He_ (14605 Å^2^) and CCS_model_ (14531 Å^2^) indicating that gas-phase IM-MS measurements of the HerA–NurA complexes are an accurate descriptor of their solution-phase molecular shapes.

In order to gain insight into the location of the bound dsDNA, we measured the ^TW^CCS_He_ of HerA–NurA with and without dsDNA. Comparison of the average ^TW^CCS_He_ between the apo-HerA–NurA (14605 Å^2^) and HerA–NurA–dsDNA (14780 Å^2^) complexes show a Δ^TW^CCS_He_ of +175 Å^2^ (1.2%). Furthermore, the apo-HerA hexamer (12230 Å^2^) and HerA–dsDNA (12375 Å^2^) similarly show a Δ^TW^CCS_He_ of +145 Å^2^ (1.2%). The increase in ^TW^CCS_He_ upon dsDNA binding is insignificant when taking into account the resolution of traveling wave ion mobility spectrometry with a total error of ±5–8% (49,50) (Figure 3D and Supplementary Figure S2E). These observations reveal that dsDNA binding induces no detectable conformational change to hexameric HerA and HerA–NurA. Moreover, from our differential collision induced unfolding (ΔCIU), we show that HerA–NurA-dsDNA complex is more stable than the HerA–NurA (Figure 3E and Supplementary Figure S3F). We therefore conclude that dsDNA occupies the inner cavity of HerA–NurA and stabilises the assembled complex.

### HerA–NurA binds a maximum of two ATPs per hexamer

**Figure 4.**
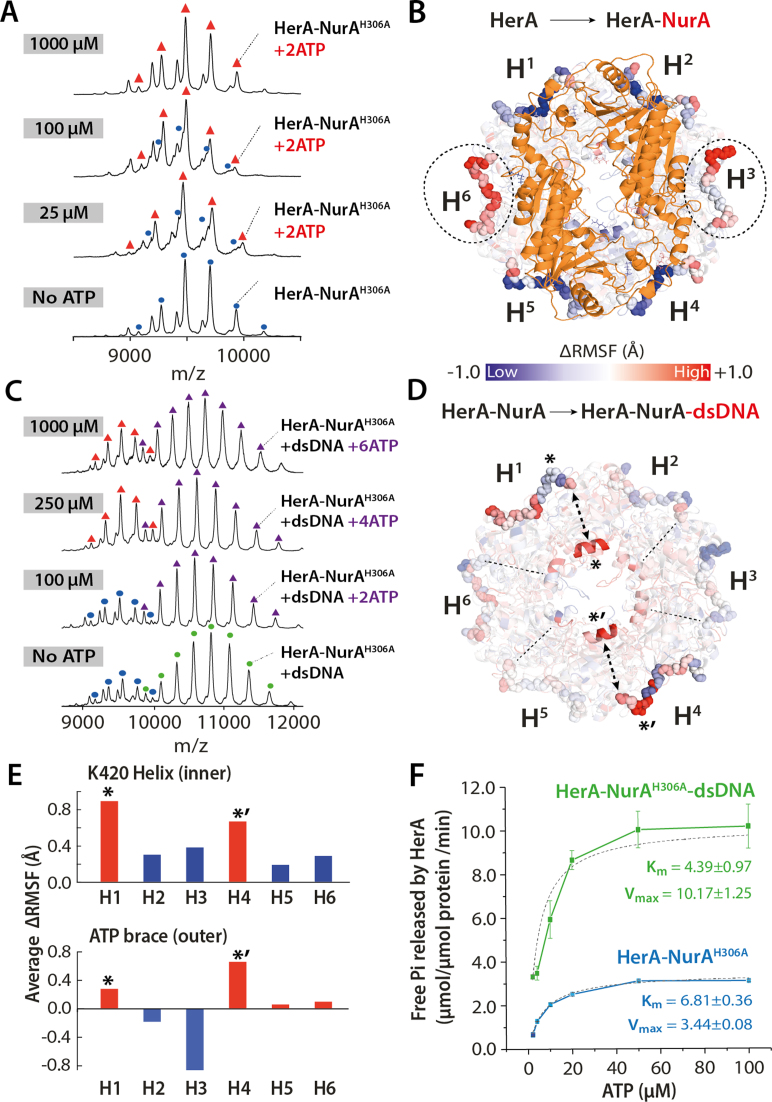
Nucleotide binding of HerA–NurA and its influence on dsDNA affinity. MS spectra of (**A**) HerA–NurA^H306A^ in the presence of increasing concentrations of ATP-γ-S. ATP-γ-S concentrations are shown for each spectra. Unassigned peaks corresponding to heptameric HerA and are removed for clarity. The titration was performed using 30μM monomeric HerA and 10μM monomeric NurA. (**B**) ΔRMSF difference map showing the effect of NurA (orange) binding to HerA over 50 ns explicit solvent simulations. Enhanced flexibility of opposite HerA ATP braces (dotted circle) in H^3^ and H^6^ subunits, is observed in the presence of NurA. (**C**) MS spectra of HerA–NurA^H306A^-dsDNA in the presence of increasing concentrations of ATP-γ-S. The presence of dsDNA enables the complex to bind two, four and six ATP-γ-S. (**D**) ΔRMSF difference map of HerA–NurA±DNA (DNA not shown) over 50ns simulation. Symmetric coupling of the internal K420 helix (opaque central helix) and external ATP brace in H^1^ and H^4^ positions are visible during DNA occupation of the HerA cavity. (**E**) Bar charts represent per-subunit average ΔRMSF of the K420 helix (top) and outer ATP brace (bottom) and show coordinated increase in flexibility in positions H^1^ and H^4^. (*) and (*’) denote the pairs of K420 helices with the external ATP braces in H^1^ and H^4^. (**F**) ATPase assay measuring the rate of ATP hydrolysis by HerA–NurA^H306A^ and HerA–NurA^H306A^-dsDNA at increasing ATP concentrations. ATPase activity at different ATP concentrations were determined by recording A_360_ over time. Experiments were performed in triplicate. The mean and the standard error of the mean are depicted (Supplementary Figures S3–S5 and Supplementary Tables S3–S5).

There is a general consensus that hexameric ATPase rings may function in either a concerted way (where nucleotide binding, hydrolysis and ADP release occurs to all subunits at the same time) or in a non-concerted fashion (where subunits bind and hydrolyze ATP at distinct times) (51,52). To examine ATP binding to the HerA–NurA^H306A^, we titrated with increasing concentrations of ATP-γ-S. Interestingly, we observed that HerA–NurA^H306A^ readily binds up to two ATP-γ-S molecules at conditions resembling cellular ATP concentrations (up to 5 mM of ATP-γ-S; Figure 4A and Supplementary Tables S3 and S4). The observation that two nucleotides readily bind to HerA–NurA^H306A^ even at a low ATP-γ-S concentration (25 μM) indicates high-affinity binding between ATP and HerA–NurA^H306A^ (Figure 4A). Furthermore, even at high ATP-γ-S concentrations (5 mM), HerA–NurA^H306A^ was unable to accommodate more than two ATP-γ-S. The two ATP-γ-S-bound state of HerA–NurA^H306A^ indicates a non-concerted mechanism of ATP binding. A reduction from six to two accessible ATP sites in HerA following association of NurA suggests conformational changes in the other four subunits leading to perturbation of ATP binding.

Next, we examined the binding of the post-hydrolysis ADP substrate to HerA–NurA^H306A^ at increasing ADP concentrations. Similar to ATP-γ-S, we observed that HerA–NurA^H306A^ binds up to two ADP at 1 mM (Supplementary Figure S3A and Supplementary Table S3). In contrast to ATP-γ-S, HerA–NurA^H306A^ binds four (250 μM ADP) and six ADP (5 mM ADP) (Supplementary Figure S3A). Whilst all ATPase sites are able to simultaneously accommodate the ADP hydrolysis product, only two ATP are able to be utilized at any one time. These results further support the notion of a non-concerted mechanism of nucleotide binding, while also demonstrating that ADP is not accompanied by the same mechanistic restrictions which exist for ATP. Together, these findings provide evidence that ATP binding to the hexameric HerA and hydrolysis within the HerA–NurA complex occurs in a coordinated pairwise manner.

While native MS revealed the discrete nucleotide binding states of HerA, it cannot distinguish which HerA subunits are simultaneously binding ATP. To extract this information and to complement the emerging mechanism of nucleotide binding, we performed explicit solvent MD simulations on the hexameric HerA and the HerA–NurA complex (Figure 4B). Interestingly, differential root-mean-squared fluctuation (ΔRMSF) analysis for HerA and HerA–NurA showed increased flexibility of the C-terminal ATP braces on opposite subunits of the hexameric ring (Figure 4B and Supplementary Figure S4). This indicates that the presence of NurA imposes two-fold symmetry on the ATP braces in opposite HerA monomers. We interpret these results as evidence of pairwise and symmetric binding of two ATP molecules to the dsDNA-free HerA–NurA complex observed in our combined native MS and MD studies.

### Binding of dsDNA to the HerA–NurA complex activates four ATP binding sites

We then set out to investigate the full complex in the presence of dsDNA and ATP. We observed that by increasing nucleotide concentrations, three different binding states corresponding to two, four and six ATP-γ-S (at 100 μM, 250 μM and 1 mM of ATP-γ-S respectively) were revealed (Figure 4C). By comparing with DNA-free experiments, we show that the presence of dsDNA opens four nucleotide binding sites, enabling up to six ATP-γ-S (1 mM ATP-γ-S; Figure 4C). This implies that the presence of dsDNA imposes local conformational changes affecting at least four HerA subunits and increases the affinity of the binding sites for ATP. Moreover, we observed four ADP bound to HerA–NurA^H306A^ –dsDNA at 1mM of ADP, while only two ADP were bound to HerA–NurA^H306A^ at the same concentrations and in the absence of dsDNA. Thus, this indicates that the addition of dsDNA sufficiently increases the affinity of HerA–NurA to ADP (Supplementary Figure S3B and Supplementary Table S3).

For four additional sites to be responsive to dsDNA loading into the inner cavity of HerA, which is distal to the ATP binding sites, indicates that the interior and exterior of HerA are mechanically coupled. MD simulations were performed in order to probe for structural changes to HerA–NurA induced by dsDNA at the atomistic level. Comparing the ΔRMSF of residues between HerA–NurA and HerA–NurA–dsDNA states, we observed coordinated flexibility between an internal HerA helix (E415–E424) and the external ATP binding brace (D461-E467) in opposite HerA subunits (Figure 4D and E and Supplementary Figure S5). Interestingly the inner helix lysine K420 projects directly into the DNA binding cavity and is the second most solvent accessible cavity lysine (SASA of 115.8 Å^2^), behind that of a known DNA interacting K363 (125.9 Å^2^). Based on our native MS and MD studies, we reason that the increased availability of binding sites from two to six ATP upon dsDNA association is due to internal-to-external coupling events induced by the binding of dsDNA and involves the K420 helix.

As dsDNA binding increases the capacity of ATP, we hypothesized that this increase would be accompanied by enhanced rates of ATPase activity. Indeed, ATPase assays show that HerA–NurA^H306A^ binds ATP with greater affinity when dsDNA bound (*K*_m_ = 4.39±0.97 μM) than unbound (*K*_m_ = 6.81±0.36 μM) (Figure 4F and Supplementary Table S5). Moreover, HerA–NurA^H306A^-dsDNA hydrolyzes ATP at a *V*_max_ of 10.17 ± 1.25 μM, approximately three-fold greater than in the apo-HerA–NurA (*V*_max_ of 3.44 ± 0.08 μM). We therefore conclude that dsDNA binding stimulates HerA–NurA to hydrolyze ATP at a much faster rate.

### DNA unwinding and degradation

The unwinding of dsDNA by helicase proteins involves translocation followed by separation of the strands in a ATP-dependent process. An ATPase reaction typically includes ATP binding, hydrolysis to ADP and inorganic phosphate (Pi), followed by dissociation in a continuous cycle. The mechanism by which the HerA ATPase drives translocation and unwinding however, has not yet been elucidated. To better understand the mechanism of action, we developed a strategy to examine the coupling between ATP and ADP binding and dsDNA processing in HerA–NurA. To do this, the nuclease-active HerA–NurA–dsDNA complex was incubated with ATP-γ-S under conditions that result in binding of two and four ATP-γ-S molecules, and then confirmed this via native MS. We then titrated with increasing concentrations of ADP and determined the number of ADP simultaneously bound to HerA–NurA–dsDNA.

**Figure 5.**
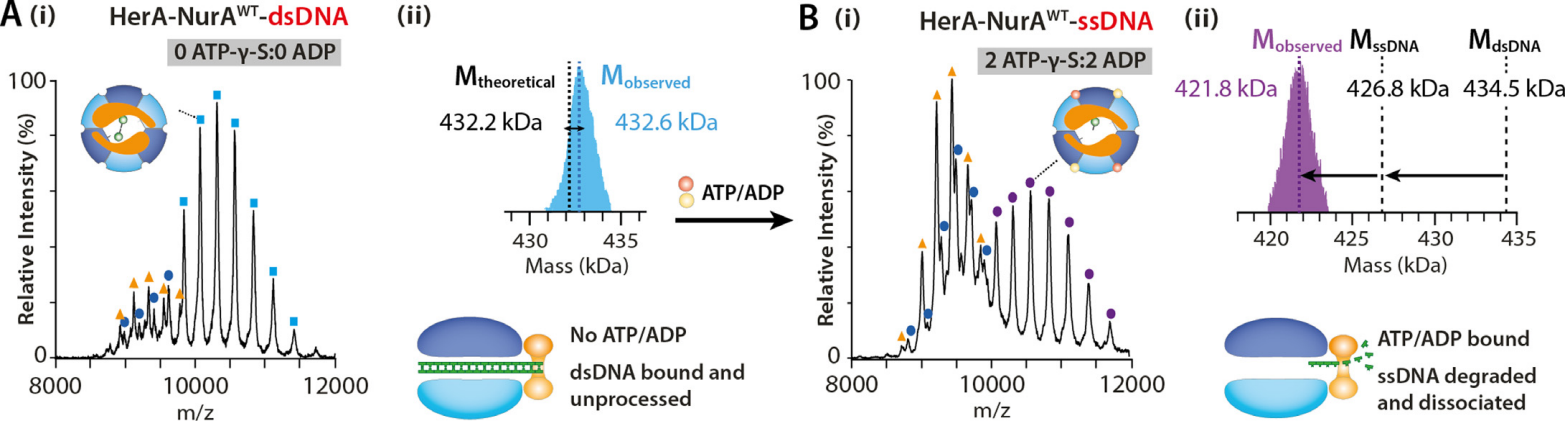
Native MS determines the mixed nucleotide binding effect on the HerA–NurA^WT^–dsDNA complexes. (**A**) (i) In the absence of ATP-γ-S or ADP, dsDNA remains bound to the HerA–NurA^WT^ complex. The apo-heptamer (orange triangle) and apo-HerA–NurA (navy circle) are shown. (ii) Transformed spectra of (i) showing observed mass (*M*_observed_) and theoretical mass (*M*_dsDNA_) of HerA–NurA^WT^–dsDNA. (**B**) Mass spectra of HerA–NurA^WT^–ssDNA in complex with 2ATP-γ-S and 2 ADP. (ii) Transformed spectra of (i) showing *M*_observed_ of HerA–NurA^WT^–ssDNA–2ATP-γ-S-2ADP compared with theoretical mass in complex with ssDNA (*M*_ssDNA_) and *M*_dsDNA_. The nuclease active HerA–NurA^WT^ processes and degrades the remaining DNA strand. As stated previously, the titration was performed using 30 μM monomeric HerA and 10 μM monomeric NurA. (Supplementary Figure S6 and Supplementary Table S3).

In the absence of nucleotides, dsDNA remains intact when bound to HerA–NurA (Figure 5A). However, for HerA–NurA bound to 2ATP-γ-S/2ADP, we measured a mass of ∼421.8 kDa (Figure 5Bi)—12.4 kDa less than the theoretical mass of HerA–NurA–dsDNA–2ATP–γ-S/2ADP (434.5 kDa; Figure 5Bii and Supplementary Figure S6). As the 25 bp dsDNA contributes ∼15 kDa of molecular weight to the HerA–NurA–dsDNA complex, the observed 12.4 kDa discrepancy would arise from dissociation of a single-strand of DNA (ssDNA) followed by partial digestion of the remaining strand. This implies that in the 2ATP-γ-S/2ADP-bound complex, the helicase and nuclease functionality of HerA–NurA is active. Binding of 4ATP-γ-S/2ADP or 2ATP-γ-S/4ADP also promotes dissociation and processing of ssDNA (Supplementary Figure S6A–C). From this, we deduce that the pairwise binding of both ATP-γ-S and ADP molecules can trigger the unwinding and dissociation of dsDNA into ssDNA. In these experiments we use a 25 bp dsDNA to understand the unwinding and dissociation process of the dsDNA and hence we can detect the ssDNA diffusing away following unwinding. However, it is important to note that *in vivo*, it is likely that the ssDNA remains tethered to the upstream unprocessed dsDNA. Similar experiments were performed with the nuclease-inactive HerA–NurA^H306A^-dsDNA mutant. We discovered that although HerA–NurA^H306A^-dsDNA similarly binds 2ATP-γ-S and ADP in pairs, native MS mass analysis confirm that dsDNA remains bound without processing by the NurA subcomplex (Supplementary Figure S6D–F). Previous efforts have shown helicase and nuclease dependent DNA degradation activities for the complex supporting our observations through MS experiments.

### DNA binds to both K363 and K420 in HerA–NurA cavity

**Figure 6.**
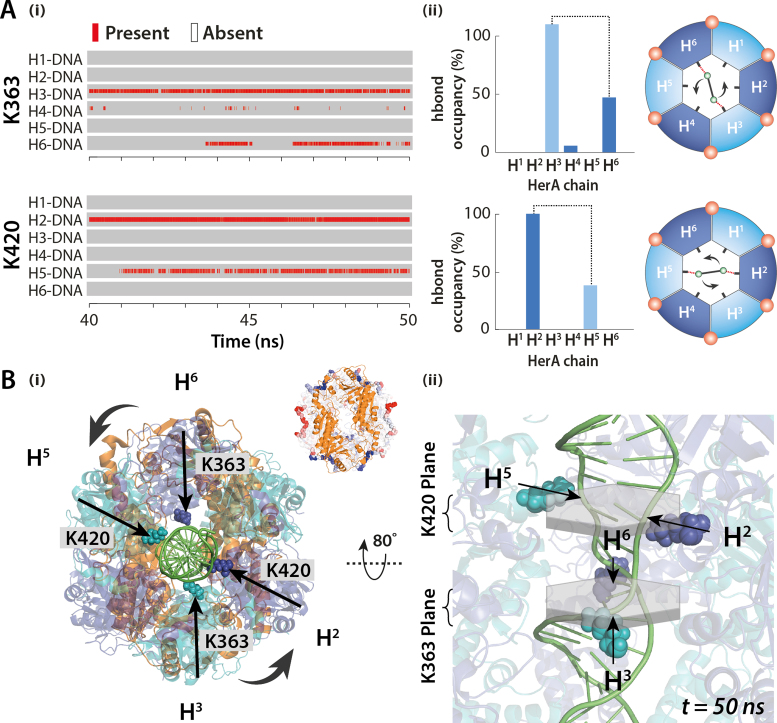
Explicit solvent MD simulations of the HerA–NurA complex bound to dsDNA and ATP. (**A**-i) Hydrogen bond interactions between K363 and K420 of HerA and DNA. HerA K363-DNA and K420-DNA hydrogen bond existence map calculated per subunit over the last 10 ns of explicit solvent MD simulations for HerA–NurA–DNA–6ATP. (A-ii) Hydrogen bond occupancy between K363 and K420 of the individual chains and the dsDNA helix. Black dotted lines indicate pairs of HerA subunits involved in coordinating dsDNA. Schematics show the interaction (dotted red lines) between DNA backbone (green) and K363 (top) and K420 (bottom) of opposite HerA subunits. ATP molecules are shown by red spheres. (**B**) Directionality of DNA backbone coordination by HerA. (i) Two pairs of K363 (H3 and H6) and K420 (H2 and H5) in four HerA subunits coordinate dsDNA. Inset highlights the 2-fold symmetry of opposite HerA ATP binding site (red loops) upon association of NurA. Same two-fold symmetry is seen from the binding of DNA to HerA K363/K420 lysines in the presence of ATP. (ii) Interior of the HerA dsDNA binding cavity. The lower plane of K363 lines coordinate the DNA backbone. Right-handed helical turn of DNA duplex is accommodated by binding to the upper plane of K420 lysines in the consecutive anti-clockwise HerA subunit (Supplementary Figure S7).

To appreciate the effect that ATP binding has on dsDNA loaded into HerA, we performed MD simulations with the HerA–NurA–dsDNA and HerA–NurA–dsDNA–6ATP complexes. Through monitoring hydrogen bond interactions between HerA and dsDNA over the simulation time, we recorded notably different interaction modes induced by ATP. When six ATP molecules occupy the binding sites of HerA, coordinated interactions between HerA residues and dsDNA were observed (Figure 6A). Hydrogen bond existence maps over the final 10 ns of simulation time were calculated between K363–dsDNA and K420–dsDNA in HerA–NurA–dsDNA–6ATP (Figure 6A).

While in association with NurA and 6ATP, HerA K363 forms contacts with opposite subunits in positions H3 and H6, K420 contacts opposite subunits in neighboring H2 and H5 (Figure 6B). However, in the absence of 6ATP, no such coordination between K363/K420 and dsDNA was observed (Supplementary Figure S7). K363 and K420 are arranged as two hexameric planes of upper (K420) and lower (K363) dsDNA interaction sites (Figure 6Bii). Remarkably, the positions of K363 and K420 in anti-clockwise consecutive subunits, complements the right-handed turn of dsDNA and results in linearization and stretching of the DNA duplex (Figure 6Bii). In contrast, no such stretching of dsDNA was observed in the absence of NurA (Supplementary Video). This supports previous evidence that there is no DNA unwinding in the absence of NurA and that the helicase activity of HerA is possible while only in the presence of NurA (7). Based on these findings, we postulate that ATP triggers the helicase activity of HerA, coordinating translocation by promoting interaction between dsDNA and anti-clockwise HerA subunits.

## DISCUSSION

Our study is the first to describe the assembly pathway and the regulation of the HerA–NurA DNA resection machinery through the interactions between the different components, namely the HerA helicase, NurA nuclease, DNA substrate, and ATP/ADP nucleotides. Furthermore, the work we present here captures several intermediates of the DNA-end resection pathway and provides mechanistic insight into how this assembly operates.

### HerA oligomeric selection

**Figure 7.**
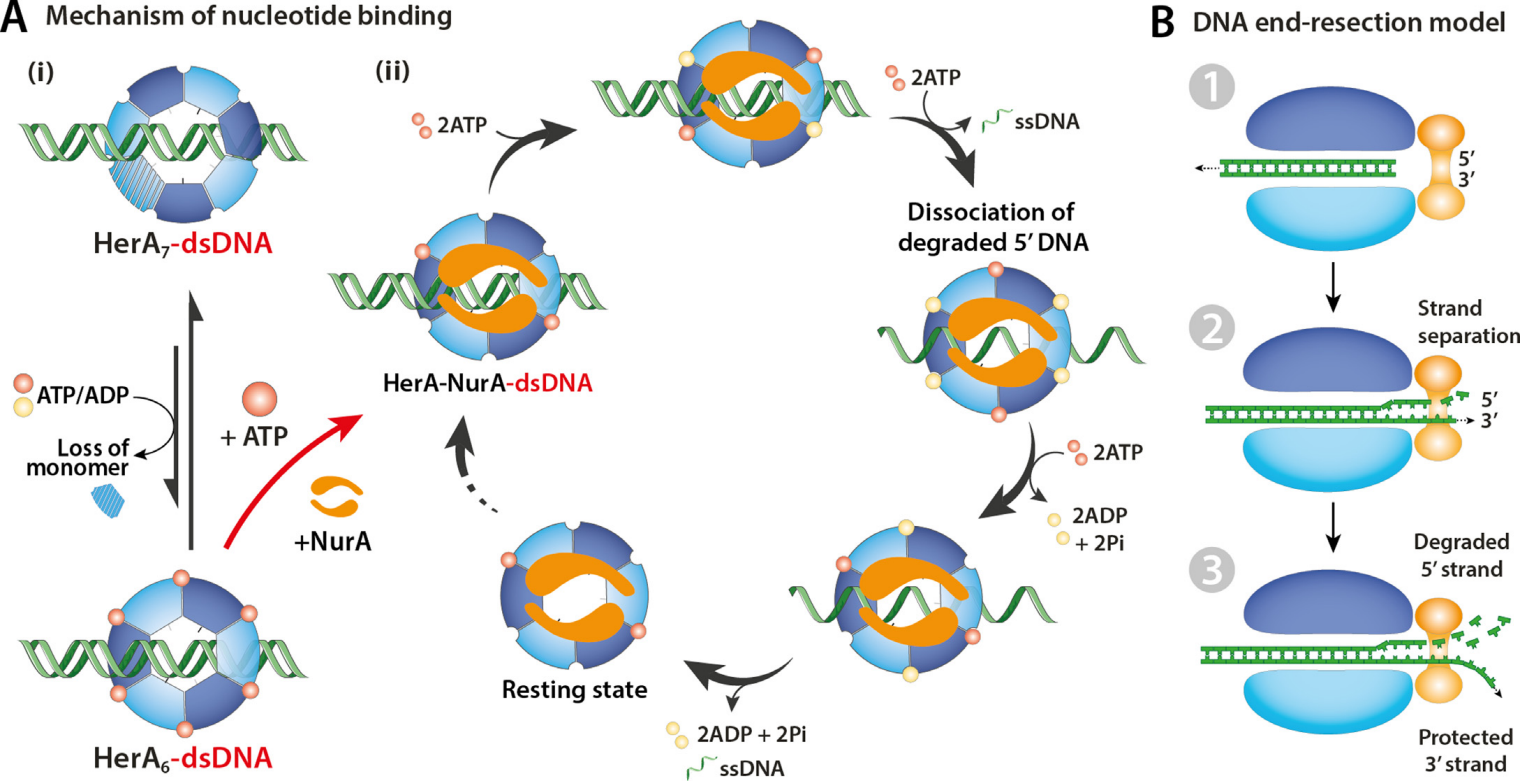
Proposed mechanism for oligomeric formation, and dsDNA and nucleotide binding of the HerA–NurA complex. (**A**) (i) HerA exists in an equilibrium between its hexameric and heptameric states. The binding of ATP or ADP favors formation of the hexamer. The NurA dimer exclusively binds to hexameric HerA, forming the HerA–NurA complex. (ii) Model showing a complete cycle of the rotational, ‘para’ mechanism of nucleotide binding, hydrolysis and ssDNA release. Each subunit alternates over ATP-bound (red spheres), ADP-bound (yellow spheres) and empty states during the catalytic cycle. (**B**) Schematic of the DNA end-resection model. (1) The dsDNA enters the complex via the pore in HerA and is translocated in an ATP-dependent manner towards NurA. (2) Following DNA unwinding and strand separation, DNA enters the channel of NurA. (3) Multiple ATP reaction cycles of HerA–NurA results in 5′ is degradation whilst the 3′ strand remains tethered to the upstream DNA.

Our results have shown that ATP-binding to HerA triggers reorganization from heptameric to hexameric rings. This conversion explains the hexameric topology of the HerA crystal structure when bound to AMP-PNP (15). The stimulation of hexamer formation by nucleotide binding has previously been reported for the bacterial enhancer-binding protein NtrC1 (53) and the Simian virus large T antigen (54). While the HerA heptamer still remains in abundance in the presence of nucleotides, this is contrary to the Bacteriophage T7 Gene 4 where the heptameric state is absent in the presence of nucleotides (55). The association of the NurA nuclease to HerA results in the oligomeric selection of the hexameric oligoform (Figure 7Ai). While both ATP and NurA show preference for the HerA hexamer, it is unclear what conformational changes may result from their association due to the lack of high-resolution structural models.

Interestingly, while the HerA heptamer is unable to bind dsDNA in the presence of nucleotides, the hexamer can bind dsDNA regardless of nucleotide binding. This indicates that ATP binding triggers different conformational responses in hexameric and heptameric forms and results in greater affinity for dsDNA only in the hexamer. The large abundance of HerA heptamer in the absence of NurA could serve multiple purposes. Firstly, due to a larger opening, heptameric HerA can better accommodate threading of dsDNA into the HerA cavity. Secondly, the heptameric HerA may be a precursor complex that facilitates loading of the HerA complex onto dsDNA, but is unlikely to have an active role in DNA processing due to lack of NurA and the inability to simultaneously bind ATP and dsDNA. Finally, the binding interface of HerA to NurA is only accessible at dsDNA ends and thus NurA may facilitate the conversion from heptamer to hexamer upon binding and initiate dsDNA processing. However, further in-depth structural studies will be required to support these hypotheses. In summary, we have uncovered the assembly mechanism of the HerA–NurA complex, consisting of hexameric HerA and the NurA dimer, and additionally proposed the role of NurA as the architectural organizer of the resection machinery.

### Nucleotides bind to HerA–NurA in pairs

Several models of nucleotide binding and exchange have been proposed for different hexameric ring–shaped ATPases, either in a concerted fashion (all-or-none) or in a non-concerted fashion (binding at distinct times) (51). Native MS showed hexameric HerA can bind six ATPs in an all-or-none manner. In contrast, we demonstrate that HerA–NurA can only bind a maximum of two ATPs. NurA tightly associates to the hexameric HerA while preserving the capacity for dsDNA binding to the central cavity. The loading of dsDNA triggers further conformational changes activating the ATP binding capabilities of the remaining subunits in a pairwise manner, leading to full saturation of the HerA ATP binding sites (Figure 7Aii). Therefore, a non-concerted mechanism involving ATP binding, hydrolysis and dissociation must be adopted by HerA in the presence of NurA and dsDNA.

### HerA–NurA possesses a concerted ‘para’ mechanism for ATP hydrolysis

There are three configurations in which a pair of ATPs can bind a hexameric ring (51): binding to adjacent subunits (ortho) (56), to subunits separated by an empty site (meta), or to opposite subunits (para) (57). To provide a visual framework for our experimental data and pinpoint the nucleotide binding modality of HerA–NurA-dsDNA we performed MD simulations. Association of NurA to HerA results in contrasting flexibility between ATP binding sites in opposite subunits, situated perpendicular to the longest dimension of NurA. We have summarized these findings in a model which shows pairwise nucleotide binding to opposite HerA in a para mechanism (Figure 7Aii). The pairing of opposite subunits is likely to be critical in converting the energy from ATP hydrolysis into mechanical work performed on the DNA substrate. Paired binding of ATP and hydrolysis in two opposite subunits provides a more efficient mechanism of force delivery and substrate interaction which can drive translocation and unwinding (56).

### Coupling ATP reaction cycle and DNA processing by the HerA–NurA complex

It has been previously shown that the nucleolytic degradation of dsDNA is dependent on the combined activities of HerA and the nuclease function of NurA (7,8). However, investigating the link between simultaneous ATP binding and hydrolysis, together with unwinding and processing of dsDNA proves difficult for structural biology techniques. Native MS experiments allowed the many heterogeneous ATP-γ-S/ADP bound states of HerA–NurA to be captured. Our MD simulations have revealed a novel K420 lysine responsible for coupling of the internal dsDNA to external ATP braces, hence coordinating dsDNA/ATP interactions. The function of K420 has not yet been described by previous studies. Furthermore, we expanded on the function of K420 which together with K363, facilitates the anti-clockwise rotation of the dsDNA substrate. Such interactions show strong association between opposite HerA subunits, providing evidence that ATP and dsDNA binding are coupled events.

Moreover, native MS has observed coordination between ATP and ADP binding. As six sites are available, pairwise binding of ATP followed by hydrolysis to ADP allows the neighboring two sites to accept new ATP in a cyclic manner. The hydrolysis of two ATP molecules into ADP and the recruitment of two additional ATPs can be reflected in the 2ATP/2ADP bound state (Figure 7Aii). In this state, ATP is hydrolyzed to ADP, followed by recruitment of two ATP and mechanical translocation and unwinding of dsDNA. Presumably many rounds of the ATP reaction cycle are necessary to process dsDNA. We have not detected DNA unwinding by HerA in isolation, or while in the presence of the nuclease-inactive NurA^H306A^. Our analyses emphasize the need for the NurA nuclease in stimulating the helicase activity for HerA (8), enabling the generation of ssDNA.

Our combined experimental and computational study supports a model of DNA unwinding, where the 3′ strand is separated from the 5′ strand upon entering the NurA nuclease. The dsDNA is recruited into the HerA–NurA cavity as confirmed by IM-MS (Step 1, Figure 7B). The helicase activity of HerA mechanically drives dsDNA towards the nuclease site of NurA (Step 2, Figure 7B). Additionally, the NurA dimer has been shown to accommodate an unwound DNA duplex (7,10). We postulate that the 3′ strand passes though the channel without undergoing any nucleolytic processing, and remains tethered to the upstream unprocessed dsDNA, making it available for homologous recombination (Step 3, Figure 7B). This is consistent with the one-strand digestion mode of DNA processing (7).

It has been suggested that the Mre11–Rad50 complex binds DNA DSB ends and aids recruitment of HerA–NurA. However, it was not known whether the Mre11–Rad50 dissociates from the processed DNA ends (58–60). The model of archaeal DNA end resection described here demonstrated that HerA and HerA–NurA can both alone associate with the DNA. It also shows that HerA–NurA follows an ordered assembly mechanism initiated by recruitment of the HerA heptamer. In eukaryotic organisms, the bloom helicase (BLM), the helicase/nuclease DNA2, the exonuclease 1 (EXO1) are involved in DNA end resection during DSB repair. Similar to archaeal organisms, the helicase BLM interacts physically with the nuclease DNA2 to perform DNA end resection. However, unlike NurA, DNA2 also comprises helicase activity, albeit dispensable (61). Moreover, cryo-EM revealed that in the absence of ATP and DNA, BLM forms hexameric ring-like structures (62). Our finding that the helicase HerA in fact predominantly exists as heptamer prior to hexamer formation and NurA nuclease recruitment may serve as a model for DNA end resection in eukaryotes.

Concluding, we have provided mechanistic insight describing the assembly pathway and nucleotide binding mechanism of the HerA–NurA complex. To date, there is no other study showcasing the many layers of conformational response and activation of HerA–NurA by ATP or by DNA association. Our detailed MD studies have identified the coupling mechanism between the dsDNA and ATP binding sites. However, determining the extent of conformational changes that accompany these coupling events will require further investigation. At present, there is no evidence as to how HerA–NurA may function together with the Mre11–Rad50 complex, responsible for downstream resection processes. Both structural and functional analysis will be invaluable to provide appropriate insight into how helicases and nucleases such as the archaeal HerA–NurA or eukaryotic Sgs1-Exo1/Dna2 collaborate with the Mre11–Rad50 during metabolic DNA processing.

Archaeal organisms maintain genome integrity despite diverse and extreme environmental stress. Therefore, archaeal proteins provide structural and mechanistic knowledge on key DNA replication and repair proteins (63). In this study, archaeal *S. solfataricus* is used as a model organism together with native MS and MD in a hybrid structural approach. This combination provides a structural framework for further study and understanding of other helicase–nuclease DNA end resection systems.

## Supplementary Material

Supplementary Data
