# Supplementary material for: Mechanistic insight into the assembly of the HerA–NurA helicase–nuclease DNA end resection complex
Source: Nucleic Acids Res. 2017 Oct 9;45(20):12025–38. doi: 10.1093/nar/gkx890 (PMC5715905; doi:10.1093/nar/gkx890)
Supplement: Supplementary Data [file gkx890_supp.zip › nar-02356-h-2017-File002.pdf]

Supplementary Material

Mechanistic insight into the assembly of the HerA-NurA helicase-nuclease DNA end resection complex

Zainab Ahdash[1], Andy M. Lau[1], Robert Thomas Byrne[2], Katja Lammens[2], Alexandra Stuetzer[3,4], Henning Urlaub[3,4], Paula J. Booth[1], Eamonn Reading[1], Karl-Peter Hopfner[2], Argyris Politis[1]*

[1] Department of Chemistry, King's College London, 7 Trinity Street, SE1 1DB, London, United Kingdom

[2] Gene Center and Department of Biochemistry, Ludwig-Maximilians-Universität München, Feodor-Lynen-Strasse 25, 81377 München, Germany

[3] Bioanalytical Mass Spectrometry Group, MPI for Biophysical Chemistry, D-37077 Göttingen, Germany

[4] Bioanalytics Group, Institute for Clinical Chemistry, University Medical Center Göttingen, D-37075 Göttingen, Germany

*Correspondence:

Argyris Politis: argyris.politis@kcl.ac.uk

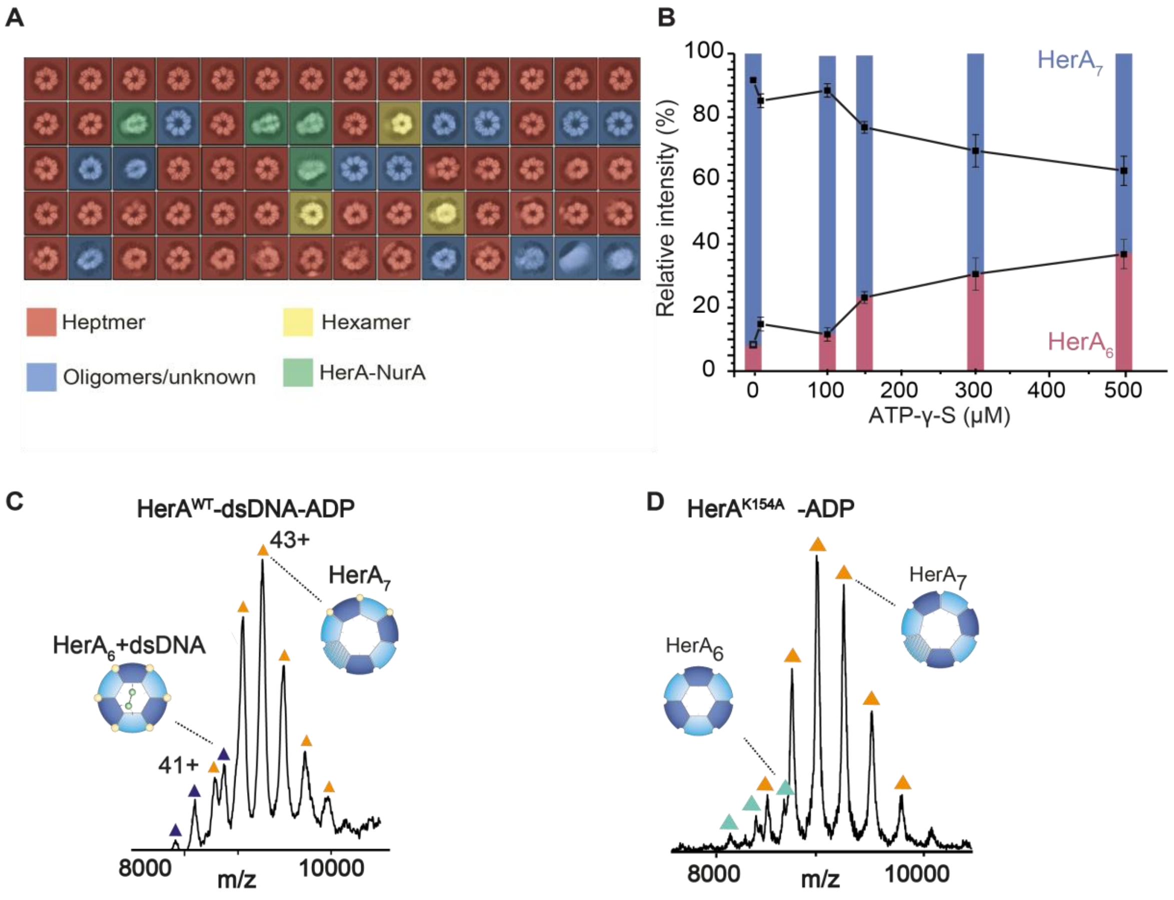

Supplementary Figure S1. Heptameric and Hexameric HerA. (A) 2D class averages grid obtained from cryo-EM showing an abundance of heptameric HerA (red) and two hexamers (yellow). Also observed are HerA-NurA and other oligomeric conformations. **(B)** Relative intensities from native MS on HerA, were extracted using UniDec deconvolution software (1). **(C)** Mass spectra of $HerA^{WT}$ in the presence of dsDNA and 1 mM ADP (**D**) Mass spectra of the lower m/z regions of $HerA^{K154A}$ incubated with 1mM ADP.

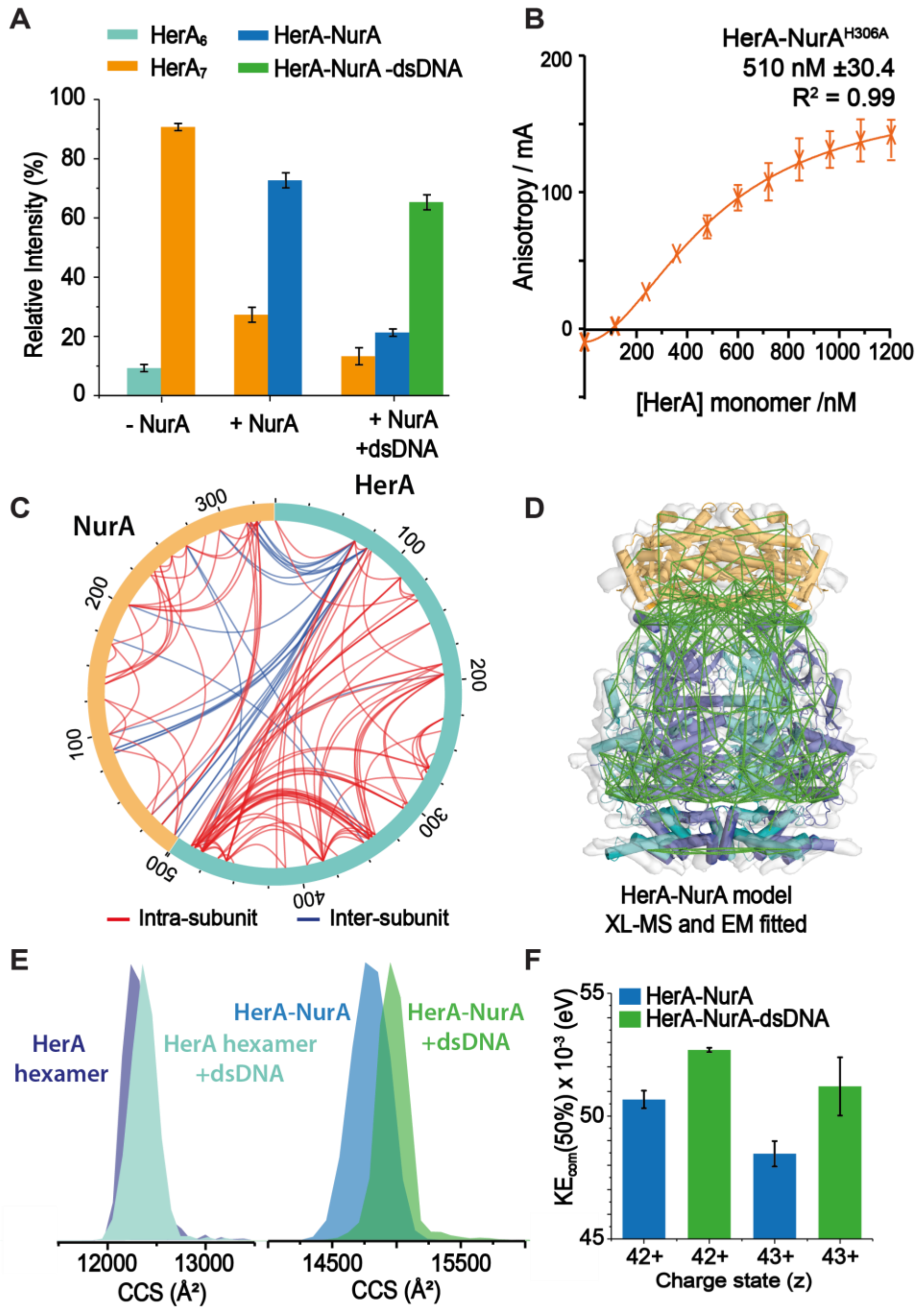

Supplementary Figure S2. NurA dimer interaction with hexameric. (**A**) Relative intensities of identified species in the absence and presence of NurA and dsDNA. **(B)** Fluorescence anisotropy assay measures the affinity of dsDNA to HerA-NurA, indicating that NurA enhances the affinity for dsDNA in comparison to HerA alone. The monomeric HerA concentrations for the complex are reported,, however NurA was present at a 6:2 ratio respectively. **(C)** Cross-linking MS identified 120 intra (red) and 23 inter-protein cross-links (blue). **(D)** Identified cross-links were projected onto

the model structure of HerA-NurA. **(E)** Accommodating the dsDNA has no significant increase in the CCS of the HerA hexamer or the HerA-NurA complex. CCS peaks for the most abundant charges are shown. HerA hexamer (40+), HerA hexamer-dsDNA (41+), HerA-NurA (44+) and HerA-NurA-dsDNA (41+). **(F)** Center-of-mass collision energies (KE_{COM}) for different charge states (42+ and 43+) show increased stability upon dsDNA binding.

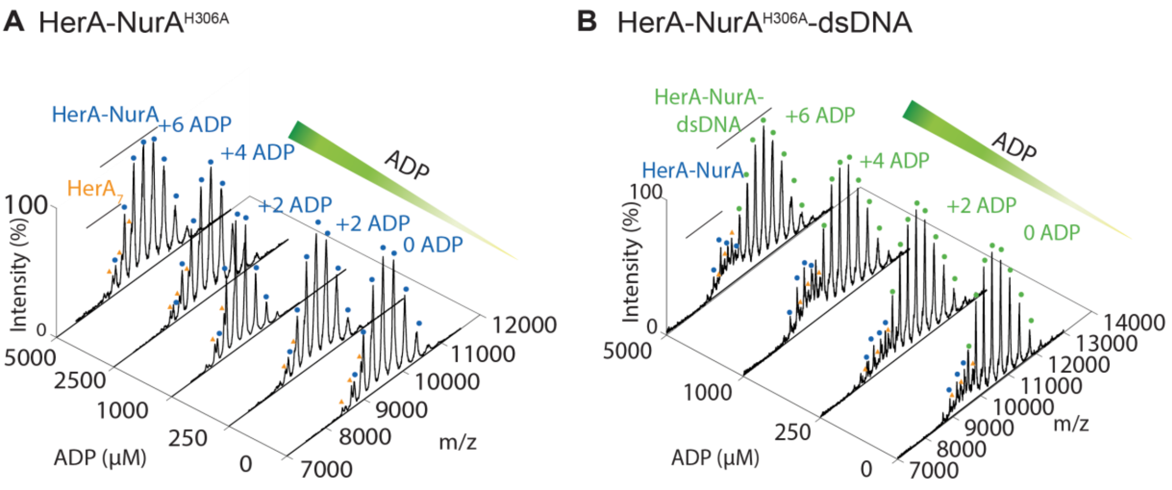

Supplementary Figure S3. ADP binding native MS experiments. Mass spectra of ADP titration to (**A**) HerA-NurA^{H306A} and (**B**) HerA-NurA^{H306A}. ADP concentrations are shown for each spectrum. The number of bound ADP molecules is noted on the spectra. The titration was performed using 30µM monomeric HerA and 10µM monomeric NurA.

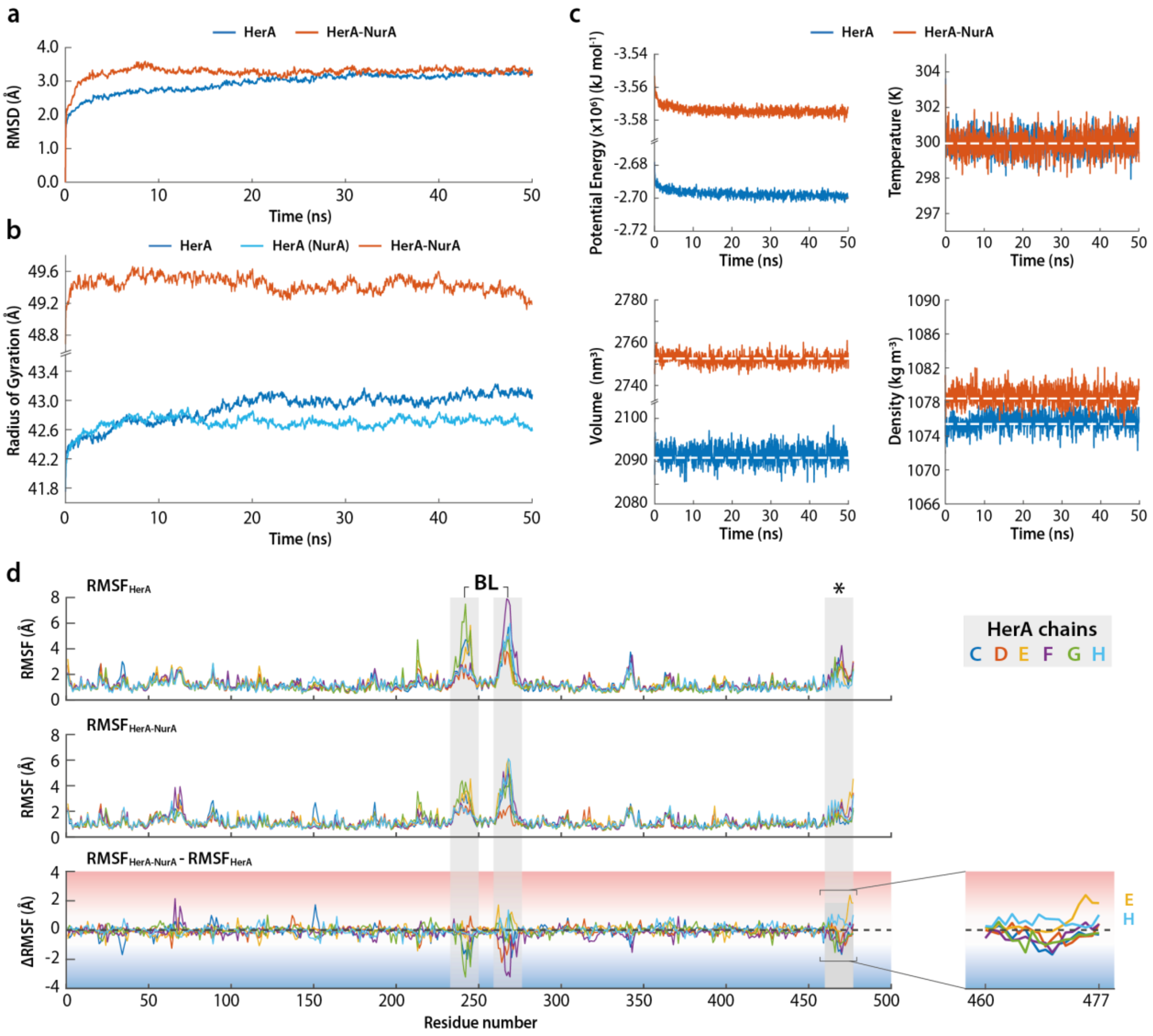

Supplementary Figure S4. Geometric and energetic parameters from simulations of apo-HerA and apo-HerA-NurA. (**A**) Root mean square deviation (RMSD) of HerA and HerA-NurA complexes over 50ns of explicit solvent simulation (every 5th frame shown). RMSD were calculated after least-squares fitting of the complete HerA and HerA-NurA complexes. (**B**) Radius of gyration of HerA alone, HerA in the presence of NurA, and HerA-NurA over 50ns (every 5th frame shown). (**C**) Global system potential energy, temperature, volume and density of HerA and HerA-NurA over 50ns. Average temperatures of 299.957 and 299.956K, volumes of 2091.43 and 2752.38nm^3 and densities of 1078.79 and 1075.66 kg m^{-3} maintained for HerA and HerA-NurA simulations respectively (dotted lines). (**D**) Per-residue

sidechain RMSF of HerA, HerA-NurA simulations and RMSF difference plot of $RMSF_{HerA\text{-}NurA}$ - $RMSF_{HerA}$. Grey boxes marked ‘BL’ represent disordered basal loops modelled onto the crystallographic structure of HerA (PDB ID: 4D2I). Grey box marked by ‘*’ depicts the crystallographically-defined C-terminal ATP brace of HerA. Inset shows magnification of ΔRMSF for each HerA subunit. Chains E and H experience greater flexibility in the presence of NurA.

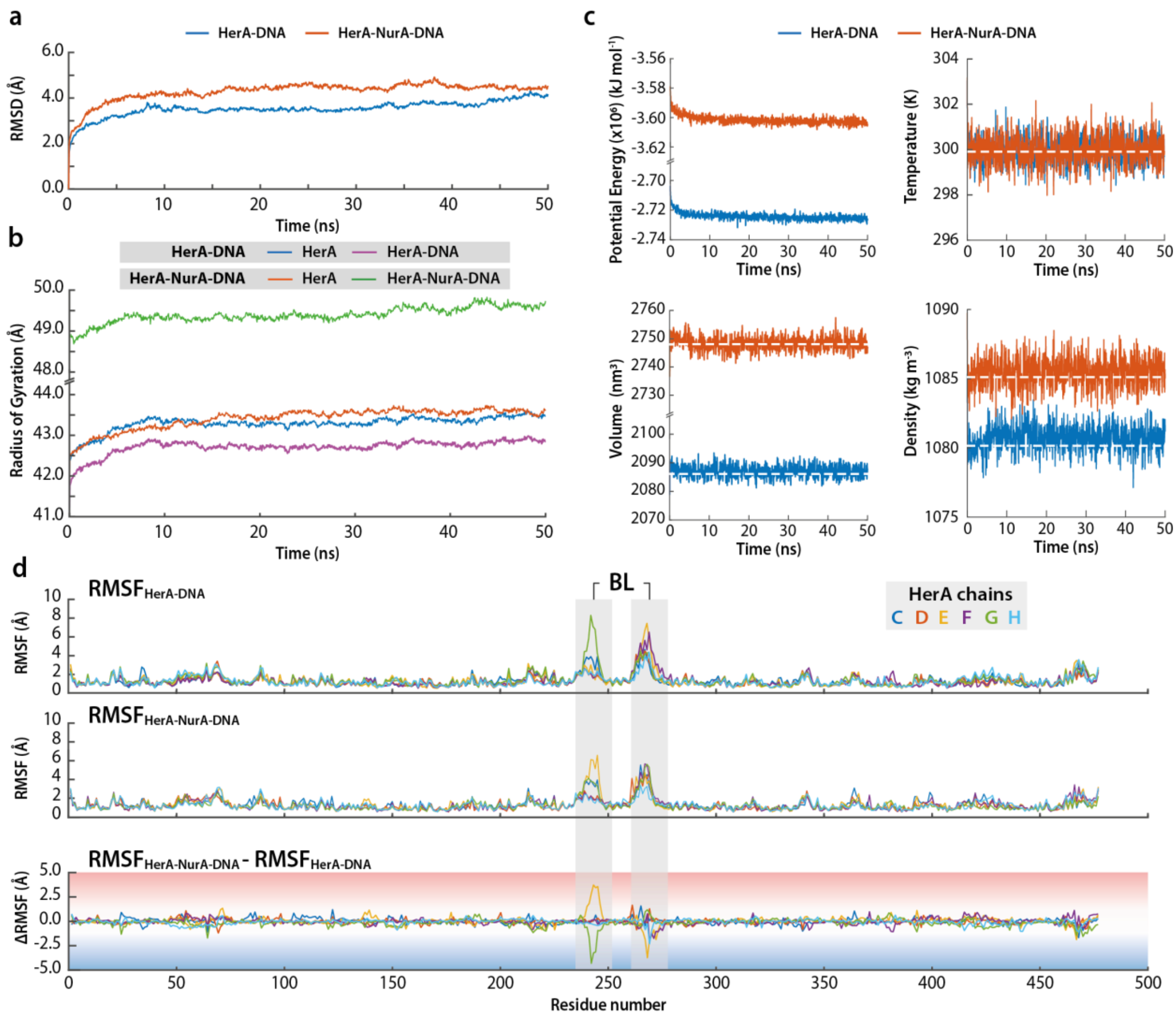

Supplementary Figure S5. Geometric and energetic parameters from simulations of HerA-DNA and HerA-NurA-DNA. (**A**) Root mean square deviation (RMSD) of HerA-DNA and HerA-NurA-DNA complexes over 50ns of explicit solvent simulation (every 5th frame shown). RMSD were calculated after least-squares fitting of the complete DNA bound HerA and HerA-NurA complexes. (**B**) Radius of gyration

of HerA alone (blue/orange) and full complexes (green/purple) over 50ns (every 5th frame shown). (**C**) Global system potential energy, temperature, volume and density of HerA-DNA and HerA-NurA-DNA over 50ns. Average temperatures of 299.953 and 299.956K, volumes of 2086.94 and 2748.46nm^3 and densities of 1085.50 and 1080.65 kg m^{-3} maintained for HerA-DNA and HerA-NurA-DNA simulations respectively (dotted lines). (**D**) Per-residue sidechain RMSF of Her-DNA, HerA-NurA-DNA simulations and RMSF difference plot of $RMSF_{HerA\text{-}NurA\text{-}DNA}$ - $RMSF_{HerA\text{-}DNA}$. Grey boxes marked 'BL' represent disordered basal loops modelled onto the crystallographic structure of HerA (PDB ID: 4D2I)

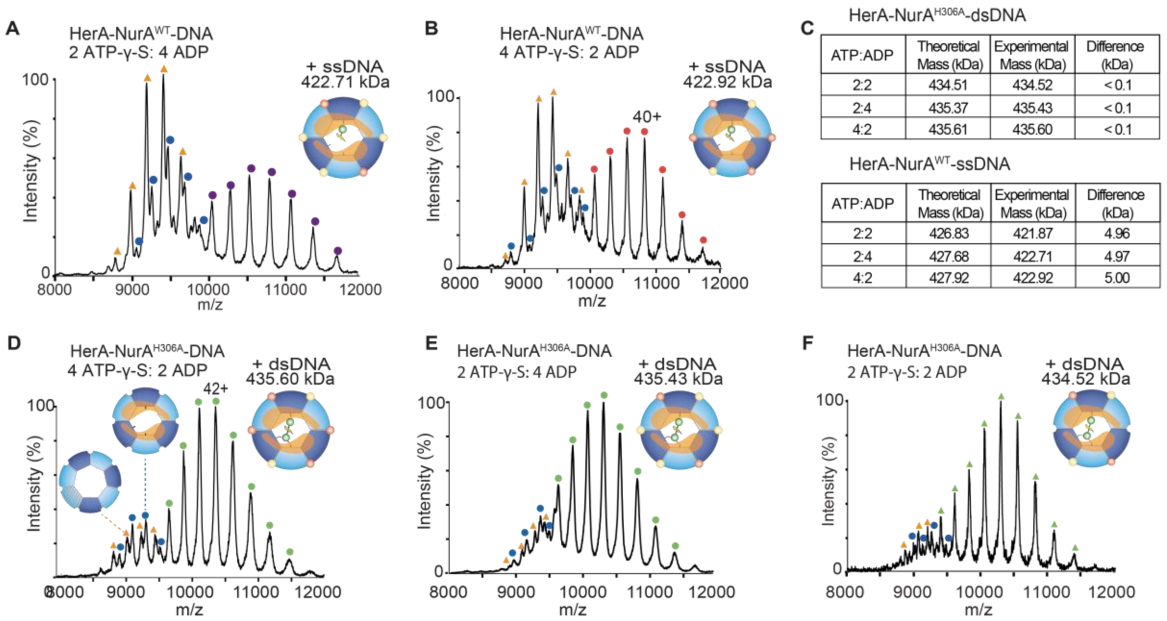

HerA-NurA^{H306A}-dsDNA

ATP:ADP	Theoretical Mass (kDa)	Experimental Mass (kDa)	Difference (kDa)
2:2	434.51	434.52	< 0.1
2:4	435.37	435.43	< 0.1
4:2	435.61	435.60	< 0.1

HerA-NurA^{WT}-ssDNA

ATP:ADP	Theoretical Mass (kDa)	Experimental Mass (kDa)	Difference (kDa)
2:2	426.83	421.87	4.96
2:4	427.68	422.71	4.97
4:2	427.92	422.92	5.00

Supplementary Figure S6. Mixed nucleotide binding states. Native mass spectra of HerA-NurA^{WT}-dsDNA in the (A) two ATP-γ-S and four ADP and (B) four ATP-γ-S and two ADP bound states. (C) Tables list the theoretical and experimental masses for mixed binding states. Native mass spectra of HerA-NurA^{H306A}-dsDNA in the (D) four ATP-γ-S and two ADP, (E) two ATP-γ-S and four ADP and (F) two ATP-γ-S and two ADP binding states. 30μM monomeric HerA and 10μM monomeric NurA were used.

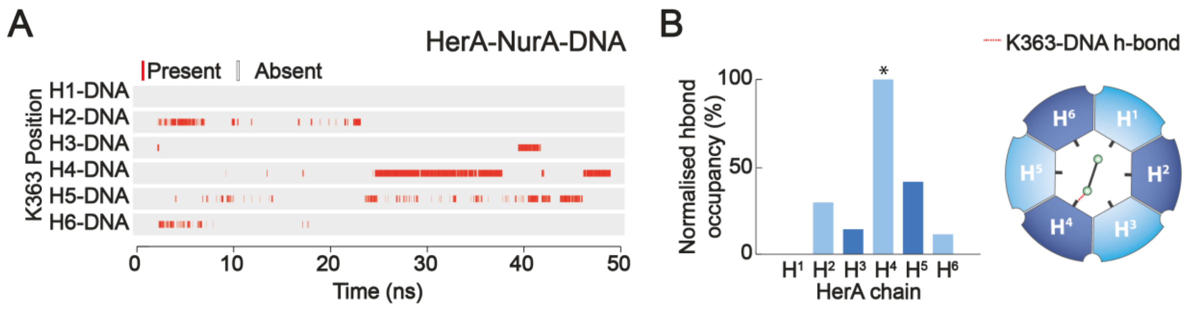

Supplementary Figure S7. Explicit solvent MD simulations of the HerA-NurA complex and dsDNA. Hydrogen bond interactions between K363 of HerA and DNA. **(A)** Per-chain HerA K363-DNA hydrogen bond existence map calculated across 50ns explicit solvent MD simulations of HerA-NurA-DNA. **(B)** Normalized hydrogen bond occupancy between K363 of the individual chains and the dsDNA helix from (middle). (*) marks the HerA chains that form major interactions with DNA. Schematics of the K363-DNA interactions of HerA-NurA-DNA in the absence of ATP molecules show non-specific to specific binding between DNA (green) and HerA monomers (blue/cyan). Interactions between K363 (black lines) and DNA are shown by red dotted lines.

Supplementary Table S1. Affinity of HerA monomer [WT] for dsDNA in the absence and presence of ATP-y-S.

	Anisotropy 1:1 binding	No nucleotide	ATP-y-S
**$HerA^{WT}$**	K_d (µM)	4.85	3.22
	Std. Error K_d	0.47	0.27
	R^2	0.98	0.98

Supplementary Table S2. Connectivity between HerA and NurA from cross-linking MS

Number	Connectivity	Protein 1	Residue 1	Protein 2	Residue 2
1	HerA-NurA	HerA	66	NurA	4
2	HerA-NurA	HerA	66	NurA	17
3	HerA-NurA	HerA	66	NurA	28
4	HerA-NurA	HerA	66	NurA	84
5	HerA-NurA	HerA	66	NurA	87
6	HerA-NurA	HerA	66	NurA	99
7	HerA-NurA	HerA	66	NurA	202
8	HerA-NurA	HerA	66	NurA	296
9	HerA-NurA	HerA	66	NurA	318
10	HerA-NurA	HerA	66	NurA	321
11	HerA-NurA	HerA	66	NurA	326
12	HerA-NurA	HerA	77	NurA	4
13	HerA-NurA	HerA	77	NurA	17
14	HerA-NurA	HerA	77	NurA	84
15	HerA-NurA	HerA	77	NurA	87
16	HerA-NurA	HerA	77	NurA	296
17	HerA-NurA	HerA	77	NurA	318
18	HerA-NurA	HerA	77	NurA	321
19	HerA-NurA	HerA	77	NurA	326
20	HerA-HerA	HerA	1	HerA	66
21	HerA-HerA	HerA	1	HerA	129
22	HerA-HerA	HerA	66	HerA	66
23	HerA-HerA	HerA	66	HerA	77
24	HerA-HerA	HerA	66	HerA	112
25	HerA-HerA	HerA	66	HerA	459
26	HerA-HerA	HerA	77	HerA	395
27	HerA-HerA	HerA	77	HerA	459
28	HerA-HerA	HerA	81	HerA	382
29	HerA-HerA	HerA	112	HerA	129
30	HerA-HerA	HerA	112	HerA	134
31	HerA-HerA	HerA	112	HerA	459

32	HerA-HerA	HerA	129	HerA	459
33	HerA-HerA	HerA	134	HerA	479
34	HerA-HerA	HerA	134	HerA	480
35	HerA-HerA	HerA	154	HerA	382
36	HerA-HerA	HerA	154	HerA	459
37	HerA-HerA	HerA	187	HerA	339
38	HerA-HerA	HerA	187	HerA	382
39	HerA-HerA	HerA	187	HerA	480
40	HerA-HerA	HerA	187	HerA	486
41	HerA-HerA	HerA	195	HerA	301
42	HerA-HerA	HerA	195	HerA	303
43	HerA-HerA	HerA	195	HerA	336
44	HerA-HerA	HerA	195	HerA	339
45	HerA-HerA	HerA	195	HerA	342
46	HerA-HerA	HerA	195	HerA	382
47	HerA-HerA	HerA	195	HerA	489
48	HerA-HerA	HerA	229	HerA	268
49	HerA-HerA	HerA	229	HerA	269
50	HerA-HerA	HerA	229	HerA	273
51	HerA-HerA	HerA	229	HerA	279
52	HerA-HerA	HerA	239	HerA	342
53	HerA-HerA	HerA	257	HerA	269
54	HerA-HerA	HerA	268	HerA	268
55	HerA-HerA	HerA	268	HerA	269
56	HerA-HerA	HerA	268	HerA	273
57	HerA-HerA	HerA	269	HerA	269
58	HerA-HerA	HerA	269	HerA	273
59	HerA-HerA	HerA	269	HerA	279
60	HerA-HerA	HerA	279	HerA	382
61	HerA-HerA	HerA	301	HerA	306
62	HerA-HerA	HerA	301	HerA	486
63	HerA-HerA	HerA	306	HerA	479
64	HerA-HerA	HerA	306	HerA	480
65	HerA-HerA	HerA	306	HerA	486
66	HerA-HerA	HerA	306	HerA	489
67	HerA-HerA	HerA	336	HerA	342
68	HerA-HerA	HerA	336	HerA	382
69	HerA-HerA	HerA	336	HerA	479
70	HerA-HerA	HerA	336	HerA	486
71	HerA-HerA	HerA	339	HerA	342
72	HerA-HerA	HerA	339	HerA	382
73	HerA-HerA	HerA	339	HerA	479
74	HerA-HerA	HerA	339	HerA	480
75	HerA-HerA	HerA	339	HerA	486

76	HerA-HerA	HerA	342	HerA	382
77	HerA-HerA	HerA	342	HerA	466
78	HerA-HerA	HerA	342	HerA	479
79	HerA-HerA	HerA	342	HerA	480
80	HerA-HerA	HerA	342	HerA	486
81	HerA-HerA	HerA	342	HerA	489
82	HerA-HerA	HerA	347	HerA	382
83	HerA-HerA	HerA	347	HerA	480
84	HerA-HerA	HerA	347	HerA	486
85	HerA-HerA	HerA	370	HerA	459
86	HerA-HerA	HerA	382	HerA	395
87	HerA-HerA	HerA	382	HerA	459
88	HerA-HerA	HerA	382	HerA	466
89	HerA-HerA	HerA	382	HerA	480
90	HerA-HerA	HerA	382	HerA	486
91	HerA-HerA	HerA	412	HerA	459
92	HerA-HerA	HerA	453	HerA	466
93	HerA-HerA	HerA	479	HerA	486
94	HerA-HerA	HerA	479	HerA	489
95	HerA-HerA	HerA	480	HerA	489
96	NurA-NurA	NurA	34	NurA	231
97	NurA-NurA	NurA	87	NurA	126
98	NurA-NurA	NurA	99	NurA	169
99	NurA-NurA	NurA	155	NurA	169
100	NurA-NurA	NurA	138	NurA	202
101	NurA-NurA	NurA	99	NurA	326
102	NurA-NurA	NurA	138	NurA	235
103	NurA-NurA	NurA	202	NurA	267
104	NurA-NurA	NurA	235	NurA	253
105	NurA-NurA	NurA	202	NurA	296
106	NurA-NurA	NurA	241	NurA	253
107	NurA-NurA	NurA	231	NurA	284
108	NurA-NurA	NurA	253	NurA	267
109	NurA-NurA	NurA	318	NurA	326
110	NurA-NurA	NurA	321	NurA	329
111	NurA-NurA	NurA	138	NurA	241
112	NurA-NurA	NurA	143	NurA	241
113	NurA-NurA	NurA	143	NurA	253
114	NurA-NurA	NurA	202	NurA	321
115	NurA-NurA	NurA	4	NurA	87
116	NurA-NurA	NurA	4	NurA	126
117	NurA-NurA	NurA	28	NurA	99
118	NurA-NurA	NurA	17	NurA	318
119	NurA-NurA	NurA	17	NurA	321

120	NurA-NurA	NurA	17	NurA	326
121	NurA-NurA	NurA	28	NurA	326
122	NurA-NurA	NurA	169	NurA	235
123	NurA-NurA	NurA	202	NurA	326
124	NurA-NurA	NurA	267	NurA	318
125	NurA-NurA	NurA	267	NurA	326
126	NurA-NurA	NurA	296	NurA	326

Supplementary Table S3. ATP-y-S and ADP binding to HerA and HerA-NurA

**HerA**			
**ATP-y-S (μM)**	**Number bound**		
0	0		
25	0		
150	6		
300	6		
500	6		
1000	6		
**HerA-NurA**			
**ATP-y-S (μM)**	**Number bound**	**ADP (μM)**	**Number bound**
0	0	0	0
25	2	150	0
100	2	250	2
500	2	500	2
1000	2	1000	2
2500	2	2500	4
5000	2	5000	6
**HerA-NurA-dsDNA**			
**ATP-y-S (μM)**	**Number bound**	**ADP (μM)**	**Number bound**
0	0	0	0
25	0	150	0
100	2	250	2
250	4	1000	4
1000	6	5000	6

Supplementary Table S4. Theoretical and experimental masses of HerA-NurA^{H306A} and HerA-NurA^{H306A}-dsDNA bound to ATP-γ-S or ADP.

	Experimental Mass (kDa)
HerA-NurA^{H306A}	417.9
HerA-NurA^{H306A} - 2 ATP-γ-S	419.1
HerA-NurA^{H306A} - 2 ADP	418.7
HerA-NurA^{H306A} - 4 ADP	419.6
HerA-NurA^{H306A} - 6 ADP	420.4
HerA-NurA^{H306A}-dsDNA	432.3
HerA-NurA^{H306A}-dsDNA -2 ATP-γ-S	433.4
HerA-NurA^{H306A}-dsDNA -4 ATP-γ-S	434.6
HerA-NurA^{H306A}-dsDNA -6 ATP-γ-S	435.7
HerA-NurA^{H306A}-dsDNA - 2 ADP	433.2

Supplementary Table S5. Activity and affinity of HerA-NurA^{H306A} and HerA-NurA^{H306A}-dsDNA for ATP.

	HerA-NurA^{H306A}	HerA-NurA^{H306A}-dsDNA
K_m (μM)	6.81 ±0.36	4.39 ±0.97
V_{max} (μM)	3.44 ±0.08	10.17 ±1.25
Std. Error K_m	0.10	0.39
R^2	0.99	0.91

Supplemental materials and methods

Molecular biology

The Walker A mutant HerA^{K154A} was produced by site-directed mutagenesis using the template *pETDuet1::SsHerA(2)* and the primers 5’-CTACTGGTTCTGGGGCGTCAAATACAGTAGC-3’ (forward) and 5’-CTACTGGTTCTGGGGCGTCAAATACAGTAGC-3’ (reverse). The nuclease-dead mutant NurA^{H306A} was produced similarly using the template *pETDuet1::SsNurA(2)* and the primers 5’-CATTTCCACTCTTAAAAGCTGCGATGGATGTTAGGTTTTCAGC-3' (forward) and its reverse complement 5’-GCTGAAAACCTAACATCCATCGCAGCTTTTAAGAGTGGAAATG-3’ (reverse). The sequences of both mutants were confirmed by Sanger sequencing (GATC Biotech). All proteins were individually produced in Rosetta pLysS (DE3) as described previously (2). For binding studies the oligonucleotide 5’-GTAGTCCGGACGACAAACGCCGACT-3’ (forward) and its reverse complement 5’-AGTCGGCGTTTGTCGTCCGGACTAC-3’ (reverse) were synthesized (Metabion) and re-suspended in water to a final concentration of 100 µM. The duplex was formed by mixing the oligonucleotides in an equimolar concentration, heating to 95°C for 5 minutes and then slowly cooling the mixture to room temperature. For fluorescence anisotropy experiments a variant of the forward oligonucleotide with a 5’-fluorescein label was used.

Electron microscopy

The grid was imaged with a Talos Arctica transmission electron microscope (FEI) with a Falcon 3EC direct electron detector (FEI) operating in counting mode. 494 micrographs were collected at a magnified pixel size of 1.002 Å/pixel between defocus values of -1.5 and -3.0 µm. The 41.77 second exposures were split into 80 frames with a dose rate of 1.0 electrons/pixel/second. The frames in each stack were motion-corrected with *motioncor2*(3) and imported into *Relion 2.0*(4) for all subsequent steps. The contrast transfer function for each micrograph was determined in *ctffind4*(5) using the unweighted sum from *motioncor2*. 80 micrographs with poorly determined CTFs were discarded and 414 were used for further analysis. The dose-weighted sum from *motioncor2* was used for all subsequent steps. 1757 particles were picked semi-interactively from 10 micrographs with *e2boxer*(6) and subjected to reference-free 2D classification in *Relion 2.0*. The best classes were then used as templates for automatic particle picking using a 40 Å low-pass filter and in total 221 680 particles were picked. The resulting reference-free 2D class averages showed predominantly top and bottom views (along the axis of the central channel in HerA) of heptameric species and a smaller number of hexa-, octa- and nonameric species. The strong orientation bias resulting absence of any side views prevented any further analysis of the data.

Gas-phase unfolding (CIU) measurements and data analysis

To test the stabilizing effect of dsDNA in the unfolding trajectory of the HerA-NurA, we subjected the HerA-NurA and HerA-NurA-dsDNA complexes to collision induced unfolding (CIU). CIU activates protein ions through increased collisions with the buffer gas in a mass spectrometer (7-9). We calculated the total internal energy of an ion using the center-of-mass collision energies (KE_{COM}) (10). A higher KE_{COM} value implies a more stable structure in the gas-phase. KE_{COM} analysis applied to various charge states revealed an average difference ΔKE_{COM} of 2.4 eV between the DNA-bound and DNA-free complexes.

For monitoring gas-phase unfolding trajectories and investigating the stability of nucleotide- binding states, we recorded IM-MS data by increasing the acceleration voltage from 10 V to 200 V in 10 V increments. The data acquired were analyzed using PULSAR (11). The software extracts IM intensities for native and unfolded ions at various collision voltages. A two-dimensional plot of the data is generated and fitted to an unfolding model. This enabled the quantification of unfolding transitions and stabilizations of protein ion (11,12). Here an unfolding transition is when a species with a known $^{TW}CCS_{He}$ value transitions to another larger $^{TW}CCS_{He}$ species. The midpoint of this transition is noted as CV_{50}, which is the value at which 50% of a specific state (in this case the assigned folded state) is depleted. The total internal energy available for the unfolding transition of the projectile ion is defined by the centre-of-mass collision energy (KE_{com}). KE_{com} is calculated from the kinetic energy and masses of the collision partners (protein ion and neutral gas) as described in Supplementary equation (1):

$$KE_{com}\ (eV) = CV_{50} \times z\ \frac{M_N}{M_N+M_{ION}} \quad \text{(eq. S1)}$$

Where z is the ion charge, M_{ION} is the mass of the protein ion and M_N is the mass of the neutral gas.

Chemical cross-linking MS

HerA-NurA complexes were reconstituted in presence or absence of DNA in HerA-NurA buffer (200 mM NaCl, 40 mM Hepes-NaOH pH 8.0). Non-hydrolyzable ATP (ATP-y-S) as added at a molar excess of 20, 50 or 200. Protein(-DNA) complexes were cross-linked with BS3 (Thermo Fisher Scientific) at molar ratios of 1:40 and 1:80 (protein:BS3) for 30 min at RT. The final protein concentration was 1 mg/mL. The reaction was quenched by addition of 100 mM Tris-HCl pH 7.9. Samples were separated by 4-12% SDS-PAGE and cross-linked species were excised from the gel and treated by in-gel digestion with trypsin as described(13). LC-MS/MS analyses were performed on a Q Exactive HF hybrid quadrupole-orbitrap mass spectrometer (Thermo Scientific) coupled to a nanoflow liquid chromatography system (1100 series, Agilent Technologies). The raw data of LC-MS/MS analysis were converted to Mascot generic format files with Proteome Discoverer 2.1.0.81 software (Thermo Scientific) and searched against a protein database by pLink 1.23 software (14)

Protein-protein crosslinks were filtered with 1% FDR followed by manual validation and removal of tandem spectra that did not show confident sequence coverage on both peptides.

Supplementary references

1. Marty, M.T., Wilcox, K.C., Klein, W.L. and Sligar, S.G. (2013) Nanodisc-solubilized membrane protein library reflects the membrane proteome. *Analytical and bioanalytical chemistry*, **405**, 4009-4016.
2. Byrne, R.T., Schuller, J.M., Unverdorben, P., Forster, F. and Hopfner, K.P. (2014) Molecular architecture of the HerA-NurA DNA double-strand break resection complex. *FEBS letters*, **588**, 4637-4644.
3. Zheng, S.Q., Palovcak, E., Armache, J.P., Verba, K.A., Cheng, Y. and Agard, D.A. (2017) MotionCor2: anisotropic correction of beam-induced motion for improved cryo-electron microscopy. *Nature methods*.
4. Scheres, S.H. (2012) RELION: implementation of a Bayesian approach to cryo-EM structure determination. *Journal of structural biology*, **180**, 519-530.
5. Rohou, A. and Grigorieff, N. (2015) CTFFIND4: Fast and accurate defocus estimation from electron micrographs. *Journal of structural biology*, **192**, 216-221.
6. Tang, G., Peng, L., Baldwin, P.R., Mann, D.S., Jiang, W., Rees, I. and Ludtke, S.J. (2007) EMAN2: an extensible image processing suite for electron microscopy. *Journal of structural biology*, **157**, 38-46.
7. Hopper, J.T. and Robinson, C.V. (2014) Mass spectrometry quantifies protein interactions--from molecular chaperones to membrane porins. *Angewandte Chemie (International ed. in English)*, **53**, 14002-14015.
8. Niu, S. and Ruotolo, B.T. (2015) Collisional unfolding of multiprotein complexes reveals cooperative stabilization upon ligand binding. *Protein science : a publication of the Protein Society*, **24**, 1272-1281.
9. Zhong, Y., Han, L. and Ruotolo, B.T. (2014) Collisional and coulombic unfolding of gas-phase proteins: high correlation to their domain structures in solution. *Angew Chem Int Ed Engl*, **53**, 9209-9212.
10. Wysocki, V.H., Joyce, K.E., Jones, C.M. and Beardsley, R.L. (2008) Surface-induced dissociation of small molecules, peptides, and non-covalent protein complexes. *Journal of the American Society for Mass Spectrometry*, **19**, 190-208.
11. Allison, T.M., Reading, E., Liko, I., Baldwin, A.J., Laganowsky, A. and Robinson, C.V. (2015) Quantifying the stabilizing effects of protein-ligand interactions in the gas phase. *Nature communications*, **6**, 8551.
12. Laganowsky, A., Reading, E., Allison, T.M., Ulmschneider, M.B., Degiacomi, M.T., Baldwin, A.J. and Robinson, C.V. (2014) Membrane proteins bind lipids selectively to modulate their structure and function. *Nature*, **510**, 172-175.
13. Shevchenko, A., Tomas, H., Havlis, J., Olsen, J.V. and Mann, M. (2006) In-gel digestion for mass spectrometric characterization of proteins and proteomes. *Nature protocols*, **1**, 2856-2860.
14. Yang, B., Wu, Y.J., Zhu, M., Fan, S.B., Lin, J., Zhang, K., Li, S., Chi, H., Li, Y.X., Chen, H.F. *et al.* (2012) Identification of cross-linked peptides from complex samples. *Nature methods*, **9**, 904-906.
